# A single-residue change in the HIV-1 V3 loop associated with maraviroc resistance impairs CCR5 binding affinity while increasing replicative capacity

**DOI:** 10.1186/s12977-015-0177-1

**Published:** 2015-06-18

**Authors:** Javier Garcia-Perez, Isabelle Staropoli, Stéphane Azoulay, Jean-Thomas Heinrich, Almudena Cascajero, Philippe Colin, Hugues Lortat-Jacob, Fernando Arenzana-Seisdedos, Jose Alcami, Esther Kellenberger, Bernard Lagane

**Affiliations:** INSERM U1108, Institut Pasteur, 75015 Paris, France; Viral Pathogenesis Unit, Department of Virology, Institut Pasteur, 75015 Paris, France; AIDS Immunopathogenesis Unit, Instituto de Salud Carlos III, 28220 Majadahonda, Madrid, Spain; Université de Strasbourg UMR7200, Illkirch, France; Univ. Paris Diderot, Sorbonne Paris Cité, Cellule Pasteur, Rue du Docteur Roux, 75015 Paris, France; Univ. Grenoble Alpes, Institut de Biologie Structurale (IBS), 38027 Grenoble, France; CNRS, IBS, 38027 Grenoble, France; CEA, DSV, IBS, 38027 Grenoble, France

**Keywords:** HIV-1, AIDS, HIV entry, HIV replication capacity, CCR5, CD4, gp120, Maraviroc, Resistance, Allosteric inhibitor

## Abstract

**Background:**

Maraviroc (MVC) is an allosteric CCR5 inhibitor used against HIV-1 infection. While MVC-resistant viruses have been identified in patients, it still remains incompletely known how they adjust their CD4 and CCR5 binding properties to resist MVC inhibition while preserving their replicative capacity. It is thought that they maintain high efficiency of receptor binding. To date however, information about the binding affinities to receptors for inhibitor-resistant HIV-1 remains limited.

**Results:**

Here, we show by means of viral envelope (gp120) binding experiments and virus-cell fusion kinetics that a MVC-resistant virus (MVC-Res) that had emerged as a dominant viral quasispecies in a patient displays reduced affinities for CD4 and CCR5 either free or bound to MVC, as compared to its MVC-sensitive counterpart isolated before MVC therapy. An alanine insertion within the GPG motif (G310_P311insA) of the MVC-resistant gp120 V3 loop is responsible for the decreased CCR5 binding affinity, while impaired binding to CD4 is due to sequence changes outside V3. Molecular dynamics simulations of gp120 binding to CCR5 further emphasize that the Ala insertion alters the structure of the V3 tip and weakens interaction with CCR5 ECL2. Paradoxically, infection experiments on cells expressing high levels of CCR5 also showed that Ala allows MVC-Res to use CCR5 efficiently, thereby improving viral fusion and replication efficiencies. Actually, although we found that the V3 loop of MVC-Res is required for high levels of MVC resistance, other regions outside V3 are sufficient to confer a moderate level of resistance. These sequence changes outside V3, however, come with a replication cost, which is compensated for by the Ala insertion in V3.

**Conclusion:**

These results indicate that changes in the V3 loop of MVC-resistant viruses can augment the efficiency of CCR5-dependent steps of viral entry other than gp120 binding, thereby compensating for their decreased affinity for entry receptors and improving their fusion and replication efficiencies. This study thus sheds light on unsuspected mechanisms whereby MVC-resistant HIV-1 could emerge and grow in treated patients.

**Electronic supplementary material:**

The online version of this article (doi:10.1186/s12977-015-0177-1) contains supplementary material, which is available to authorized users.

## Background

The entry of human immunodeficiency virus type 1 (HIV-1) into host cells starts with the binding of the surface subunit (gp120) of the viral envelope glycoprotein (Env) to cell surface CD4. This triggers conformational rearrangements in gp120 that allow it to interact with a coreceptor, either CC chemokine receptor 5 (CCR5) or the CXC chemokine receptor 4 (CXCR4) that are G-protein coupled receptors [1]. Elements in gp120 critical for coreceptor binding comprises the third variable loop V3 and a four-stranded bridging sheet shaped from the V1/V2 stem and the C4 region [2–4]. The bridging sheet and the base of V3 are thought to interact with the N-terminus domain of the coreceptor, while the tip of V3 interacts with its second extracellular loop (ECL2) [5–8]. Interaction of gp120 with the coreceptor then leads to exposure of the transmembrane subunit (gp41) of Env, which inserts into the host cell plasma membrane and entails the viral fusion process [6, 9, 10]. Two entry inhibitors are currently used for treatment of HIV infection, i.e. the fusion inhibitor enfuvirtide (T20) and the CCR5 ligand maraviroc (MVC) (for review see Ref. [11]).

MVC belongs to a class of small molecule CCR5 inhibitors acting via an allosteric mechanism [12]. The compound binds to a CCR5 transmembrane cavity distinct from the binding sites for chemokines and gp120 and changes the coreceptor conformation in such a way that HIV/CCR5 interactions are impaired [13–15]. Resistance to MVC has been reported both in vitro and in vivo and results from viruses that have acquired the ability to use MVC-bound CCR5 in addition to free CCR5 for entry into cells [16–19]. This is manifested by maximal percents of infection inhibition (MPI) that are less than 100% at a saturating inhibitor concentration, with MPI values that decrease with increased abilities of resistant viruses to use the inhibitor-bound receptor relative to free CCR5 [19, 20]. Resistance to allosteric inhibitors has mapped to sequence changes in the V3 loop, making the virus to interact with CCR5 regions whose conformation is spared by the inhibitor (e.g. the N-terminus) [17–19, 21–24]. Resistance could also occur with no V3 changes and involve mutations in gp41 or the CD4-binding site of gp120 [25–28], suggesting that alterations of either of the different steps in HIV entry may compensate for impaired interactions with inhibitor-bound CCR5.

Acquisition of resistance to allosteric inhibitors can result in viruses that have a reduced replicative capacity, thereby leading to resistance mutations that revert rapidly when treatment with the inhibitor is discontinued [24, 29, 30]. In contrast, other resistant viruses showed no fitness loss [31]. In some cases, inhibitors can select for resistant viruses showing a reduced infectivity in some particular cells such as macrophages or central memory CD4^+^ T cells (T_CM_ cells), suggesting that continued treatment with those inhibitors might be beneficial for some patients even in the context of virological failure [16, 32]. This, unfortunately, is not always the case as improved infectivity of a MVC-resistant HIV-1 has recently been described in T_CM_ cells in the presence of the inhibitor [33].

Resistance to CCR5 inhibitors and replicative capacity are thought to be closely related to the ability of viruses to bind to entry receptors, in particular to CCR5 in its inhibitor-bound conformation. To date however, information about the binding affinities to CD4 and CCR5 for inhibitor-resistant HIV-1 remains scarce. Recently, the development of the 293-Affinofile receptor affinity profiling system has provided important clues on the relative efficiencies of CD4 and CCR5 usages for viral entry (for review see Ref. [34]). In particular, high efficiency of CCR5 usage (i.e. low CCR5 dependence) has in many cases been correlated to high level of resistance to CCR5 inhibitors, making it a possibility that the level of resistance is related to the virus ability to bind to inhibitor-bound CCR5 [17, 32]. However, given that CCR5 may contribute to different steps of HIV entry (e.g. interactions with CD4, formation of the fusion pore, triggering of signaling pathways), the extent to which a virus is dependent on CCR5 could have nothing to do with CCR5 binding affinity. The same seems also to be true for replicative capacity. Indeed, previous works showed that the extent to which fusion and replication are inhibited by CCR5 ligands may not be correlated to inhibition efficiency of Env/CCR5 interactions [8, 14, 35].

Here, we combined binding assays with purified gp120, virus infections in target cells with varying receptor expression levels, virus-cell fusion assays and molecular dynamics simulations to investigate the CD4 and CCR5 binding properties of a MVC-sensitive and a MVC-resistant Envs. Results revealed that the MVC-resistant Env has a severely impaired ability to engage both receptors in the absence and in the presence of MVC, as compared to the MVC-sensitive Env. In particular, the V3 loop of the MVC-resistant Env contains an uncommon insertion of an Alanine within the highly conserved GPGR motif (G310_P311insA), which we identified to be responsible for the decreased CCR5 binding affinity while, nonetheless, allowing the virus to use CCR5 efficiently and thereby increasing its fusion and replication efficiencies. This study thus highlights unsuspected mechanisms whereby HIV could develop resistance to CCR5 allosteric inhibitors and evolve as a dominant viral *quasispecies* in patients.

## Results

**Figure 1:**
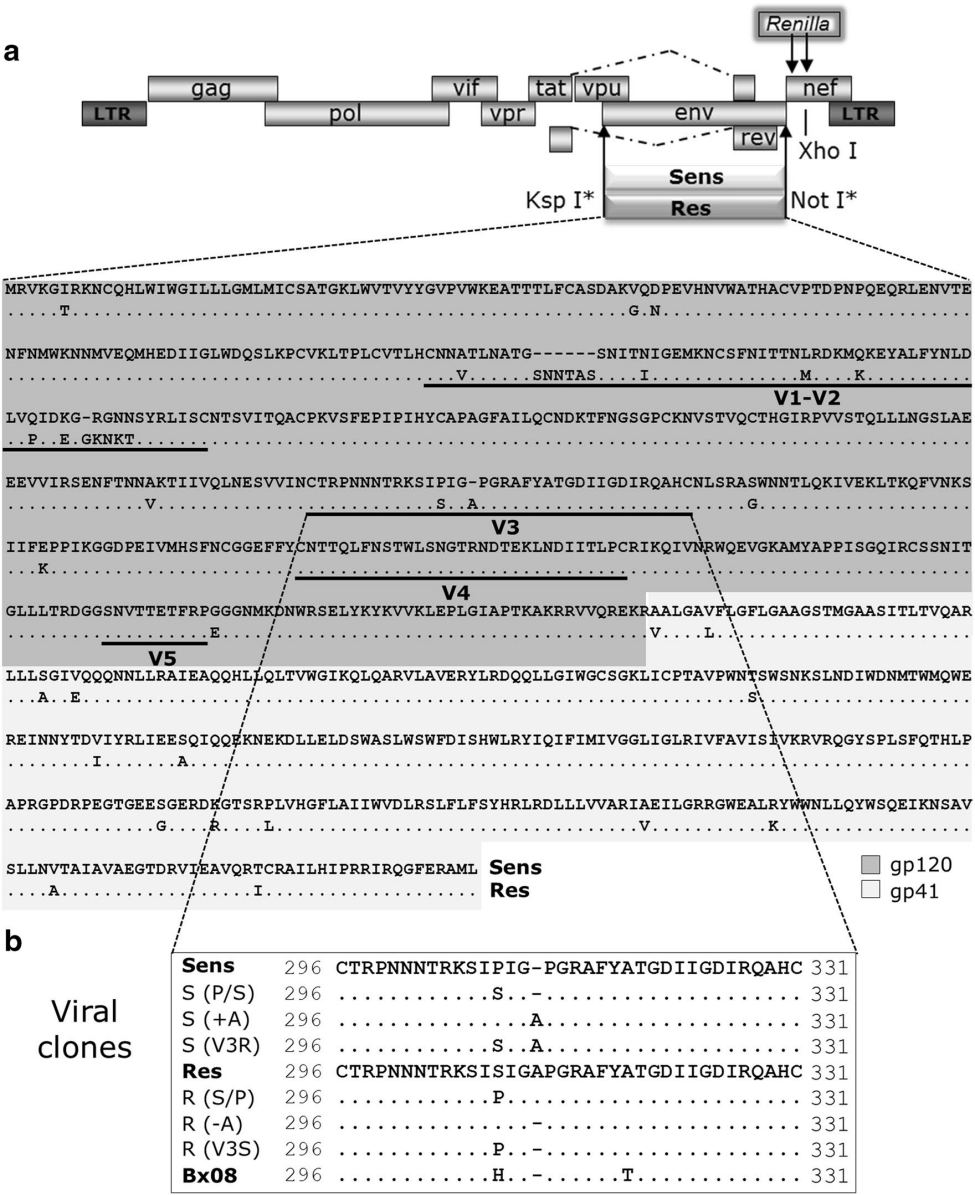
Cloning, sequence analysis and site-directed mutants of MVC-Sens and MVC-Res Envs. **a** Schematic representation of the proviral vector pNL-KspI/env/NotI-Ren. The KspI site was introduced in the proviral clone pNL4-3Ren to allow the cloning of MVC-Sens and MVC-Res gp160. Analysis of the MVC-Res Env sequence shows 32 mutations as compared to MVC-Sens Env, 18 within gp120 and 14 within gp41, as well as eight amino acid insertions within gp120. The V3 loop of MVC-Res Env contains two changes, the P308S mutation and an insertion of an Alanine within the GPGR tip (G310_P311insA). The MVC-Sens and MVC-Res Env sequences are similar to those reported in two previous papers, except in the N- and C-terminal parts where we noted several amino acid changes. Indeed, in the sequences used in the references [17] and [33], which are deposited in the Los Alamos HIV Sequence Database, the 41 first residues and the 105 last residues originate from the HxB2 HIV-1 strain. **b** Amino acid sequences of the V3 loops of the different site-directed mutants of MVC-Sens and MVC-Res used in this study. *S* and *R* refer to the parental sequences from which the mutant sequences are derived. *Dots* indicate residues that are identical to those of the parental Env sequence, and *dashes* indicate gaps. The sequence of the V3 loop of gp120 from the HIV-1 strain Bx08, to which MVC-Sens and MVC-Res Envs are compared in this study, is also shown. The first Cys residue of the V3 loop is equivalent to C296 in the HXB2 sequence and thus noted as such in the MVC-Sens, MVC-Res and Bx08 V3 sequences.

The MVC-sensitive and MVC-resistant isolates we used here (hereafter referred to as MVC-Sens and MVC-Res) represent the dominant circulating viruses isolated from a patient of the MOTIVATE clinical trial before and after MVC therapy, respectively (Pfizer INC, NY, personal communication). Analysis of the MVC-Res Env sequence shows 32 mutations as compared to MVC-Sens Env, as well as eight amino acid insertions (Figure 1). Our Env sequences are similar to those reported in two previous papers [17, 33], except in the N- and C-terminal regions where we noted several amino acid changes (see the legend of Figure 1 for more details). The V3 loop of MVC-Res Env contains two changes, the P308S mutation and the Ala insertion within the GPGR motif (G310_P311insA), which were described to be necessary for MVC resistance in NP2-CD4/CCR5 cells [17, 33]. However, whether other regions of the resistant Env play a role as well as the individual contributions of the two changes within the V3 loop in the phenotypic properties of MVC-Res have not been investigated.

### Genetic-phenotypic relationships of the MVC sensitive and MVC resistant Envs

**Figure 2:**
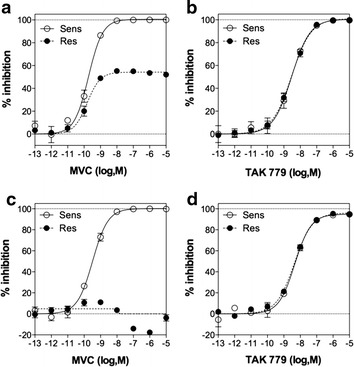
Susceptibility of MVC-Sens and MVC-Res to inhibition by MVC and TAK 779. U87-CD4/CCR5 cells (**a**, **b**) or PBMCs (**c**, **d**) were inoculated with equal amounts of MVC-Sens or MVC-Res (10 ng of Gag p24) in the absence or in the presence of increasing concentrations of MVC (**a**, **c**) or TAK 779 (**b**, **d**). *Data points* are expressed as percent inhibition of infection relative to control infection measured in the absence of MVC (0%) and were fitted to a sigmoidal dose–response model with a variable slope. Representative experiments out of five independent experiments performed in triplicate are shown.

**Figure 3:**
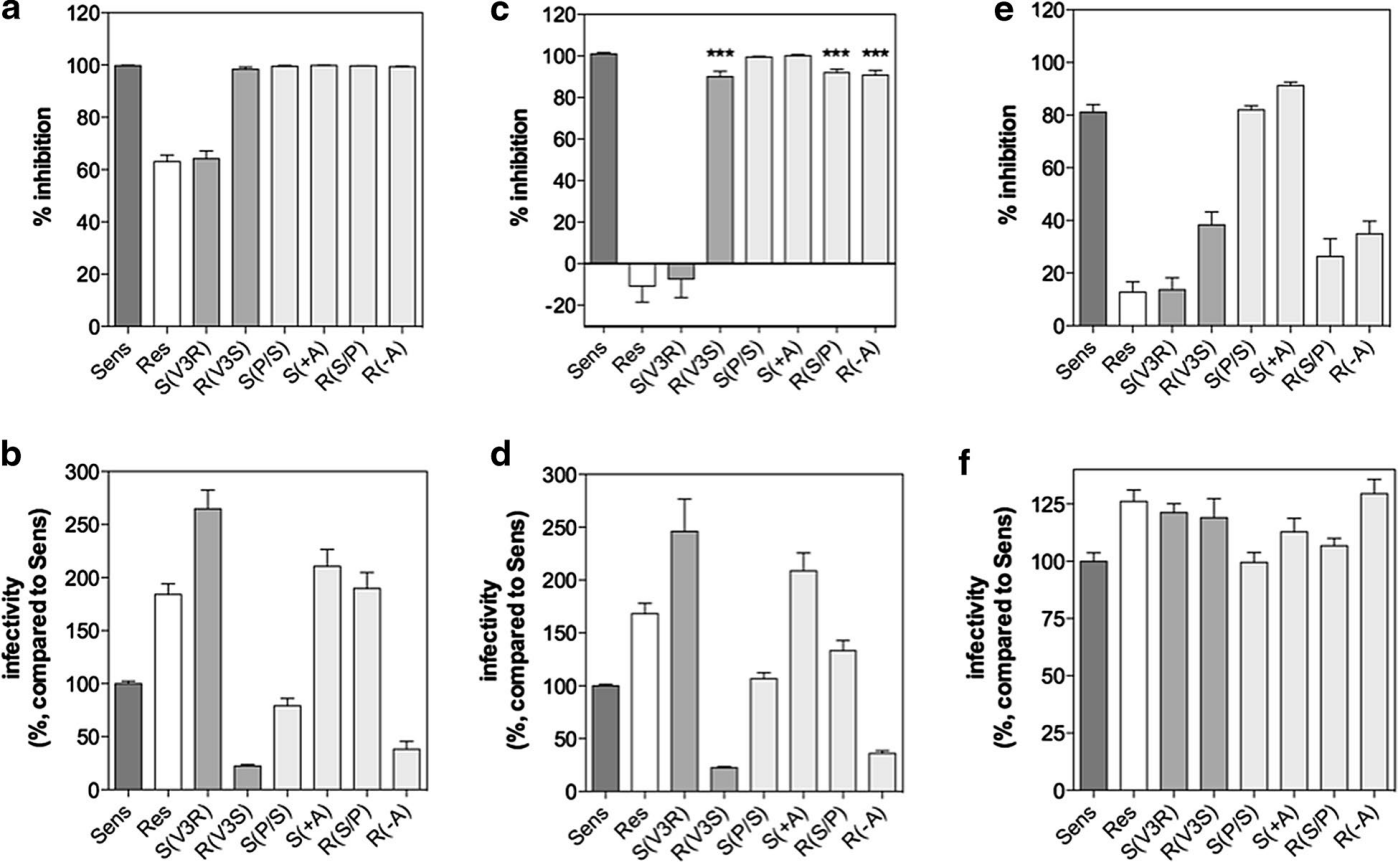
MVC resistance and viral replicative capacity: The effects of amino acid changes in the MVC-Sens and MVC-Res V3 loops and dependence on the cell type. U87-CD4/CCR5 cells (**a**, **b**), PBMCs (**c**, **d**) or CD4- and CCR5-expressing HEK 293T cells (**e**, **f**) were infected with equal amounts (10 ng of Gag p24) of MVC-Sens, MVC-Res or their related variants S(V3R), R(V3S), S(P/S), S(+A), R(S/P) or R(−A), in the absence or in the presence of 10 μM MVC. The percents of infection inhibition (panels **a**, **c**, **e**) were determined as indicated in the legend of Figure 2. Viral infectivities (**b**, **d**, **f**) were determined by measuring luciferase activity in the cell lysates 30 h (U87, HEK) or 48 h (PBMCs) post-infection and are expressed as percent infectivity relative to that of MVC-Sens (100%). Results are mean ± SEM of 3–8 independent experiments performed in triplicate. ***P < 0.001 in unpaired two-tailed Student *t* test.

As the first step to study the mechanisms of MVC resistance, we cloned the sequences encoding MVC-Sens and MVC-Res Envs into the proviral vector pNL-KspI/env/NotI-Ren derived from the pNL4-3Ren viral clone [36] to produce replication-competent viruses (Figure 1). Then, we first performed MVC resistance assays in U87-CD4/CCR5 cells, which are typically used in the PhenoSense™ Entry assay for assessment of HIV-1 resistance to CCR5 entry inhibitors [19]. At 30 h post infection in the presence or absence of increasing MVC concentrations, cell lysates were examined for their luciferase activity as readout for viral entry. Viruses expressing MVC-Sens Env were fully inhibited by MVC, while incomplete inhibition of MVC-Res Env was apparent at saturating MVC concentrations, with a mean MPI value of 63 ± 12% (see Figure 2a for a representative experiment and Figure 3a). This value is lower than those of most MVC-resistant viruses from subjects failing therapy identified using the PhenoSense™ assay (MPI > 80%). This is indicative of MVC-Res Env having a high level of resistance to MVC, in agreement with previous observations [17, 33]. We found no cross-resistance of the MVC-resistant virus to another low molecular weight, allosteric inhibitor of CCR5 (TAK 779) using the U87-CD4/CCR5 cells (Figure 2b). Substituting the V3 loop within MVC-Sens Env by that of MVC-Res Env [MVC-Sens(V3R)] conferred resistance to 10 μM MVC, while the mutant of MVC-Res with the V3 loop of MVC-Sens Env [MVC-Res(V3S)] was fully sensitive to the drug (Figure 3a), in agreement with previous work [17]. When considered individually, none of the two V3 changes present in MVC-Res Env led to MVC resistance (Figure 3a). This suggests that the combination of the two changes within V3 is necessary for MVC resistance in U87-CD4/CCR5 cells.

In PBMCs, the MPI values revealed that MVC had more modest effects on the infectivity of MVC-Res and even slightly increased it in some cases (mean MPI = −10.8 ± 34%), depending upon the individual’s PBMCs used (Figures 2c, 3c). In contrast, MVC-Sens remained fully sensitive to inhibition by MVC. The MVC-resistant virus modestly resisted to TAK 779 (MPI = 95%), but similarly to MVC-Sens (Figure 2d). The MVC-Sens(V3R) variant resisted to 10 μM MVC as efficiently as MVC-Res, as in U87-CD4/CCR5 cells, but unexpectedly, the reverse mutant [MVC-Res(V3S)], as well as MVC-Res bearing only either of the two V3 changes, i.e. the P308S mutation [MVC-Res(−A)] or the Alanine insertion in the GPG motif [MVC-Res(S/P)], also consistently showed slight levels of resistance to the drug in PBMCs as indicated by MPI values close to 90% (Figure 3c). In the context of the MVC-Sens Env, however, the individual V3 changes did not confer resistance. These results suggested that regions of the MVC-Res Env outside the V3 loop cause a low-level of MVC resistance, which is further magnified by the two changes within the V3 loop. To further confirm this conclusion, we then performed additional experiments on HEK cells expressing higher levels of CCR5 (HEK-CD4/CCR5 cells), as compared to U87-CD4/CCR5 cells and PBMCs [14]. Indeed, previous results showed enhanced resistance of HIV-1 to CCR5 allosteric inhibitors upon increasing the CCR5 expression levels at the cell surface [37–40]. In HEK-CD4/CCR5 cells, MVC-Res appeared highly resistant to 10 μM MVC (MPI = 12.7 ± 12%), whilst MVC-Sens was largely inhibited by the inhibitor (MPI = 81.2 ± 8.7%) (Figure 3e). In agreement with what the results in PBMCs had suggested, the MVC-Res-derived variants MVC-Res(V3S), MVC-Res(−A) and MVC-Res(S/P) partly resisted inhibition by MVC (26.4% < MPI < 38.3%), in contrast to the MVC-Sens(P/S) and MVC-Sens(+A) variants, which remained as sensitive as MVC-Sens to the inhibitor. Overall, these results confirm that MVC-Res, but not MVC-Sens, carries sequence motifs out of the V3 loop that confer basal resistance to MVC.

**Figure 4:**
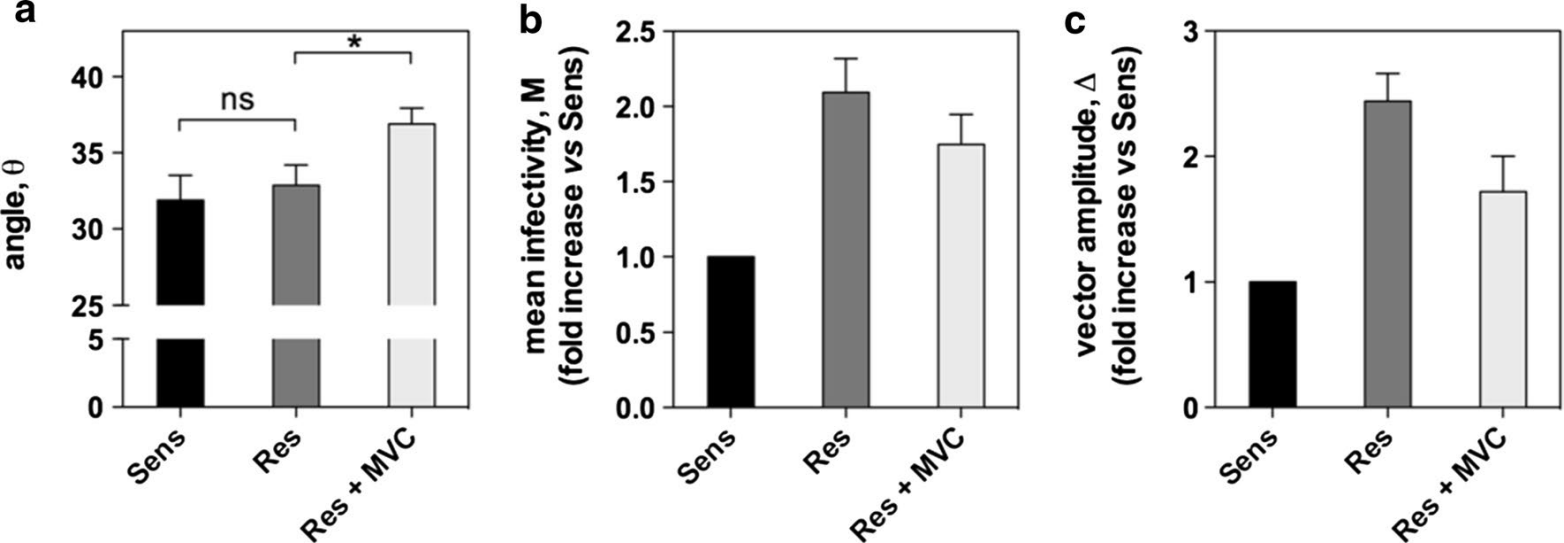
Relative efficiencies of CD4 and CCR5 usage by MVC-Sens and MVC-Res Envs as assessed by the 293-Affinofile receptor affinity profiling system. 293-Affinofile cells were induced by minocycline and/or ponasterone A to express 25 different combinations of CD4 and CCR5 expression levels and then infected by equal amounts (10 ng of Gag p24) of MVC-Sens or MVC-Res in the presence or in the absence of 10 μM MVC. Thirty hours post-infection, luciferase activity was measured in the cell lysates and the three metrics θ (**a**), M (**b**) and Δ (**c**) describing viral infectivity were then determined using the VERSA website (http://versa.biomath.ucla.edu). The maximally induced levels of CD4 and CCR5 were 287,000 and 83,000 receptor/cell. Results represent the mean ± SE of at least four independent determinations. *P < 0.05 in unpaired two-tailed Student *t* test.

As mentioned in the introduction, escape viruses to CCR5 antagonists can show changes in their replication capacity. Here, we observed that the replication of MVC-Res is increased by ≈2-fold in U87-CD4/CCR5 and PBMCs, as compared to MVC-Sens (Figure 3b, d), and is only faintly diminished in the presence of MVC (Figure 4). Results further showed that this phenotype was attributable to the Alanine insertion within the V3 loop of MVC-Res Env. Indeed, inserting Alanine in the context of the MVC-Sens Env [MVC-Sens(V3R) and MVC-Sens(+A) variants] enhanced replication by up to 2.5-fold, while removing Alanine in the resistant virus [MVC-Res(V3S) and MVC-Res(−A) variants] resulted in a 5- to eightfold loss of the extent of replication (Figure 3b, d). This indicates that changes in MVC-Res Env outside the V3 loop has caused a fitness loss, which was compensated for by the Alanine insertion. Interestingly, these differences in the replication capacities were no longer apparent in HEK-CD4/CCR5 cells expressing high levels of CCR5 (Figure 3f). This suggests that the reduced replication of viruses lacking Alanine could be rescued under conditions where CCR5 expression is high and thus that Ala plays a role in allowing viruses to use CCR5 more efficiently.

The observation that the Ala insertion confers a replicative advantage to MVC-Res was somewhat unexpected, given that we found no virus containing this insertion in the Los Alamos HIV Sequence Database. We thus suspected that the Ala insertion might have detrimental effects on replication in different Env contexts. In line with this hypothesis, we found that inserting Alanine in the V3 loop of the HIV-1 strains Bx08 and JR-CSF lowered replication to almost undetectable levels (Additional file 1: Figure S1). This strongly suggests that sequence motifs that are common to MVC-Sens and MVC-Res Envs, but that are probably absent in most of other Env contexts, have permitted the Ala insertion to increase viral replication.

In the following sections, we next investigated whether resistance of MVC-Res to MVC as well as its increased replicative capacity could be related to changes in the interactions between gp120 and CD4 or CCR5 and/or in env-mediated fusion kinetics.

### Binding characteristics of MVC-Sens and MVC-Res Envs to CD4 and CCR5

The 293-Affinofile cells wherein CD4 and CCR5 expressions can be independently and simultaneously induced have widely been used for assessing efficiencies of CD4 and CCR5 usage (for review see Ref. [34]). Typically, Affinofile cells are infected at varying amounts of CD4 and CCR5 and results are mathematically fitted to a 3D-surface function describing the viral isolate’s infectivity features. These include the overall entry efficiency or mean infectivity M, as well as a sensitivity vector whose direction (as indicated by the value of the vector angle θ) and steepness or amplitude Δ onto the surface plot indicate the relative dependence of the virus on CD4 and CCR5 and the overall rate of responsiveness to changes in CD4 and CCR5 expression levels, respectively. We found that MVC-Sens and MVC-Res had comparable θ values of 31.9 ± 1.3° and 32.9 ± 1.6°, respectively, indicating that both viruses were slightly more responsive to changes in CD4 levels than in CCR5 levels (Figure 4). We also observed that MVC-Res had increased M and Δ values reflecting higher infectivity responses, as compared to MVC-Sens Env (Figure 4). In the presence of 10 μM MVC, MVC-Res showed a significant increase in θ (37° ± 1.2°) combined with decreases in the M and Δ values, indicating that MVC-Res uses the drug-bound form of CCR5 less efficiently than the free receptor.

**Figure 5:**
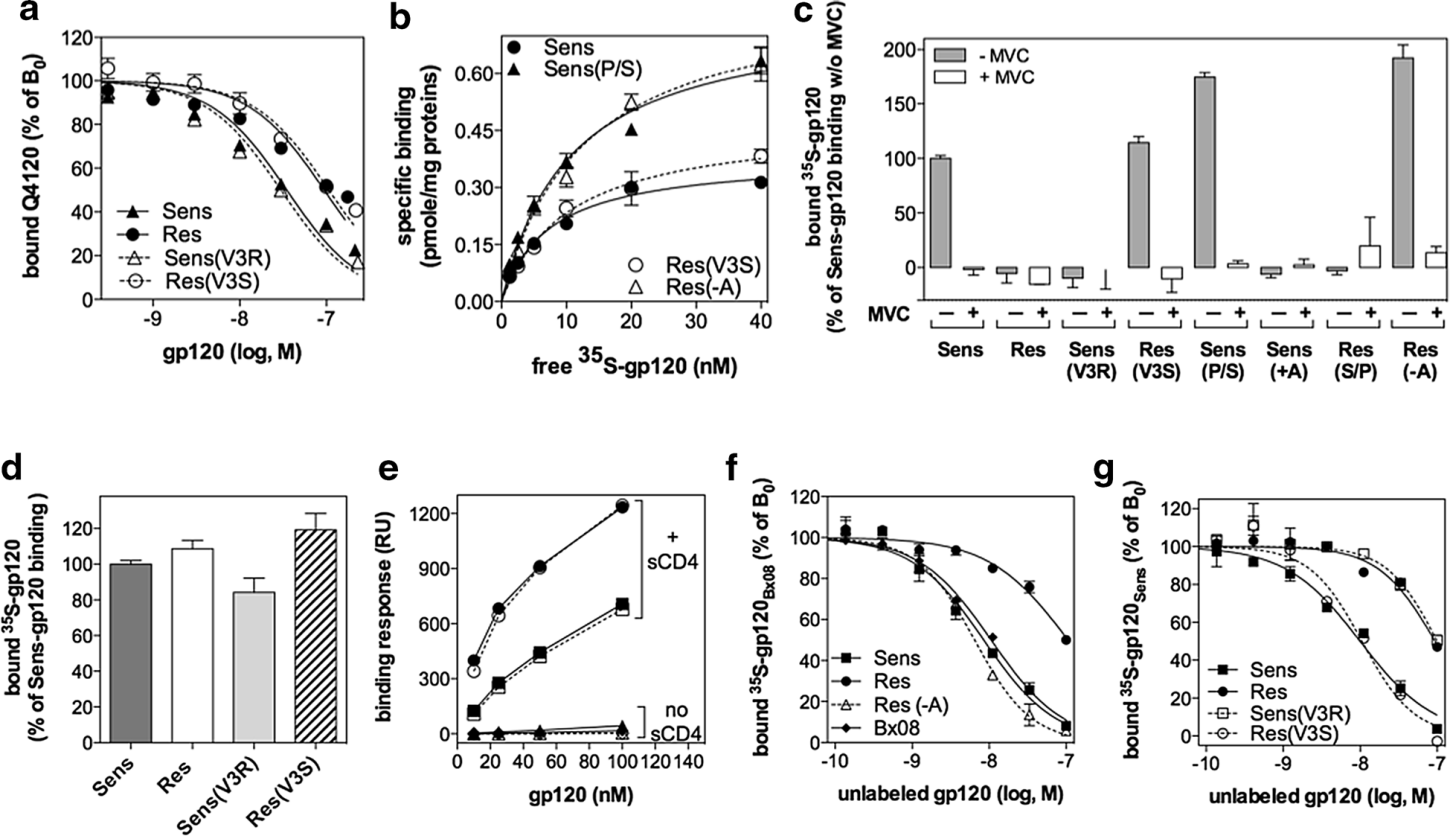
Receptor binding properties of wild-type and modified gp120 monomers derived from the MVC-Sens and MVC-Res viral isolates. **a** Competition of mAb Q4120 binding to CD4-expressing HEK 293T cells by increasing concentrations of the indicated purified monomeric gp120. Results were normalized for nonspecific binding (0%) and specific binding in the absence of glycoprotein (100%, B_0_) and were fitted to a one-site competitive binding model. A representative experiment performed in duplicate is shown (n = 3). **b** Equilibrium saturation binding of the ^35^S-labeled gp120 of MVC-Sens, MVC-Sens(P/S) (i.e. MVC-Sens wherein Pro-308 is substituted by Ser), MVC-Res(V3S) (i.e. MVC-Res whose V3 loop is replaced by that of MVC-Sens) or MVC-Res(−A) (i.e. MVC-Res lacking the Ala insertion). *Curves* represent specific binding of glycoproteins to crude membranes from CCR5-expressing HEK 293T cells, determined in the presence of 400 nM sCD4, and obtained by subtracting from total binding the non specific binding measured in the presence of 10 μM TAK779 or using parental HEK cells. Data were fitted to a one-site binding model. Representative experiments performed in duplicate are shown (n = 3–4). **c** Specific binding of 10 nM of the indicated ^35^S-labeled gp120 monomers (+400 nM sCD4) to CCR5-expressing HEK cells, in the presence (+) or absence (−) of 10 μM MVC, was calculated as in panel (**b**), and then expressed as percent of MVC-Sens gp120 binding in the absence of MVC (100%). The binding of glycoproteins measured in the presence of 10 μM TAK779 was considered as nonspecific binding in these experiments. Of note, in some cases, glycoproteins showed levels of binding that came slightly lower than this nonspecific binding, explaining why “negative” specific binding are plotted in the panel. Results represent mean ± SD of 2–5 independent experiments performed in duplicate. **d** The panel represents specific binding on CD4-expressing HEK cell membranes of the indicated ^35^S-gp120 used at a concentration equal to their K_i_ value for CD4 deduced from the displacement experiments of mAb Q4120 binding shown in panel (**a**) (see text). One experiment out of two is shown. **e** Binding of the indicated concentrations of MVC-Sens (*closed symbols* and *straight lines*) or MVC-Res (*open symbols* and *dashed lines*) gp120 to 17b (*squares* and *diamonds*) or E51 (*circles* and *triangles*) mAbs immobilized on a CM4 sensorchip, alone (*triangles* and *diamonds*) or after preincubation with 200 nM sCD4 (*circles* and *squares*). Of note, *triangles* and *diamonds* are superimposed at the bottom of the panel due to marginal binding in the absence of sCD4. *Open* and *closed squares* are also superimposed due to similar binding of MVC-Sens and MVC-Res gp120s to mAb 17b. **f** and **g** Competition of 10 nM ^35^S-gp120_Bx08_ (**f**) or ^35^S-gp120_Sens_ (**g**) binding to CCR5-expressing membranes by increasing concentrations of the indicated unlabeled gp120 was carried out in the presence of an excess concentration of sCD4 (1,000 nM). Results were normalized for nonspecific binding determined in the presence of 10 μM TAK779 (0%) and specific binding in the absence of competitor (100%, B_0_) and were fitted to a one-site competitive binding model. Representative experiments out of 2–3 independent experiments run in duplicate are shown.

To get further insights into the relative abilities of MVC-Sens and MVC-Res Envs to engage CD4 and CCR5, we next performed equilibrium binding experiments of monomeric soluble gp120 to CD4- or CCR5-expressing intact cells or cell membrane preparations. In particular, MVC-Sens, MVC-Res, MVC-Sens(V3R) and MVC-Res(V3S) gp120 were assayed for their ability to displace the binding of the anti-CD4 monoclonal antibody Q4120 to CD4 stably expressed at the surface of HEK 293T cells (HEK-CD4 cells) (Figure 5a). Indeed, mAb Q4120 binds to the gp120-binding site on domain 1 of CD4 [41]. We determined in preliminary saturation binding experiments that mAb Q4120 binds to CD4 with a dissociation constant K_D_ of 0.4 ± 0.2 nM, a value close to that reported in a recent study [42], and then used mAb Q4120 at this concentration in the subsequent competition binding assays. We found that the different gp120 variants inhibited mAb Q4120 binding with the following equilibrium dissociation constant (K_i_) values: MVC-Sens gp120 (K_i_ = 15.5 ± 2.1 nM), MVC-Res gp120 (K_i_ = 43.6 ± 4.1 nM), MVC-Sens(V3R) gp120 (K_i_ = 12.5 ± 2.6 nM) and MVC-Res(V3S) gp120 (K_i_ = 41.5 ± 12.5 nM) (Figure 5a). These K_i_ values are within the range of those reported in the literature [43, 44]. These results indicate that MVC-Res gp120 has a threefold lower affinity for CD4 than MVC-Sens gp120. They also show that substituting the V3 loop of MVC-Sens gp120 by that of MVC-Res gp120, and vice versa, do not modify binding affinities to the receptor. This is in agreement with previous data showing that discontinuous regions of gp120 outside the V3 loop contribute to CD4 binding [45].

To measure the affinity of glycoproteins for CCR5, we first performed saturation binding experiments of ^35^S-labeled MVC-Sens gp120 or MVC-Res gp120 to membranes from HEK-CCR5 cells in the presence of an excess concentration of soluble CD4 (400 nM) (Figure 5b). Specific binding of ^35^S-MVC-Sens-gp120 to CCR5 could be described as a hyperbolic function, from which we deduced a K_D_ value of 7.2 ± 1.1 nM, which is within the range of affinity constants reported for other R5 gp120 [46–48], and a maximum number of binding sites (B_max_) of 0.3 ± 0.08 pmol/mg of protein. Unexpectedly, similar experiments carried out with MVC-Res gp120 yielded levels of binding that was indistinguishable from nonspecific binding determined in the presence of 10 μM TAK779 or using membranes from parental HEK 293T cells, regardless of the presence or absence of MVC (Figure 5c). Identical results were obtained when binding was measured on intact HEK-CCR5 cells. Subsequent experiments showed that insertion of Alanine into the V3 loop of MVC-Res gp120 was accountable for its impaired ability to bind to CCR5 (Figure 5c). Indeed, introducing Alanine within the V3 loop of MVC-Sens gp120 [MVC-Sens(+A)], as well as replacing the V3 loop within MVC-Sens gp120 by that of MVC-Res [MVC-Sens(V3R)], diminished binding to CCR5 to a level similar to that observed for MVC-Res gp120, while removing this Alanine residue in MVC-Res gp120 [MVC-Res(−A)], or replacing the V3 loop of MVC-Res gp120 by that of MVC-Sens [MVC-Res(V3S)], fully restored binding to the coreceptor (Figure 5c). Of note, in contrast to binding to CCR5, we controlled that the ^35^S-labeled glycoproteins MVC-Sens, MVC-Res, MVC-Sens(V3R) and MVC-Res(V3S) bind to similar extents to CD4 (Figure 5d). This was confirmed by additional binding experiments to CD4 in which the ^35^S-gp120 were used at a concentration equal to their K_i_ value determined from the experiments presented in Figure 5a, i.e. a concentration that is expected to bind half the CD4 molecules. As shown in Figure 5d, comparable levels of binding were found for the glycoproteins under these conditions.

Saturation experiments of gp120 binding also showed that the nature of the amino acid at the V3 position 308 influences the B_max_ value, that is the maximum number of receptors to which the gp120 can bind. In particular, higher B_max_ values were measured for MVC-Sens(P/S) and MVC-Res(−A) gp120 (B_max_ = 0.53 ± 0.2 and 0.79 ± 0.14 pmol/mg, respectively) containing a Ser residue instead of Pro, as compared to MVC-Sens and MVC-Res(V3S) (B_max_ = 0.3 ± 0.08 and 0.36 ± 0.1 pmol/mg, respectively) (Figures 5b, c). This finding is reminiscent of previous results showing that different mAbs to distinct CCR5 epitopes bind to different proportions of CCR5 molecules at the cell surface [8, 49, 50]. Those results were interpreted in terms of CCR5 existing in different conformations/forms, which are differentially recognized by the mAbs. Similarly, it could be that the nature of the amino acid at position 308 influences the epitopes of CCR5, and, in turn, the coreceptor population, to which gp120 binds.

One explanation of why we were unable to detect binding of ^35^S-MVC-Res gp120 to CCR5 might be that the protein does not properly fold and/or expose the bridging sheet upon binding CD4. To investigate this possibility, we performed surface plasmon resonance experiments to measure the binding of MVC-Sens and MVC-Res gp120s to mAbs 17b and E51. These mAbs belongs to a group of monoclonal antibodies known as “anti-CD4i” which bind to conserved elements of gp120 induced by CD4 and overlapping the bridging sheet and the base of V3 making interactions with the CCR5 N-terminus [4, 51]. In the absence of sCD4, MVC-Sens and MVC-Res gp120s showed negligible binding to mAbs (Figure 5e). In contrast, after preincubation of gp120s with sCD4, binding to mAbs was dramatically induced, indicating that sCD4 has triggered conformational changes in both MVC-Sens and MVC-Res gp120s leading to the formation of the bridging sheet. Both gp120s exhibited similar levels of binding, suggesting that they have comparable affinities for the mAbs and that they have undergone similar CD4-induced conformational changes.

Despite that, the apparent lack of binding of ^35^S-MVC-Res gp120 to CCR5 made it possible that the glycoprotein has an overall decreased affinity for the coreceptor, presumably as a result of impaired V3 loop/CCR5 ECL2 interactions. To investigate this issue, we next tested the ability of unlabeled gp120 to compete for binding to CCR5 with either ^35^S-MVC-Sens-gp120 or ^35^S-gp120 from the R5 HIV-1 primary strain Bx08, which show comparable affinity for CCR5 (Ref. [14] and Figure 5f). From displacement of ^35^S-gp120_Bx08_ binding by unlabeled MVC-Sens gp120 (Figure 5f), we calculated a K_i_ value of 4.5 ± 0.2 nM for the MVC-sensitive Env, which is similar to the K_D_ value determined using the saturation binding experiments shown in Figure 5b. A K_i_ value of 6.3 ± 0.9 nM was similarly calculated for unlabeled gp120_Bx08_. In striking contrast, MVC-Res gp120 only partly displaced ^35^S-gp120_Bx08_ binding in the range of the concentrations used, with an estimated K_i_ value ten-fold higher than that of MVC-Sens (K_i_ = 50.1 ± 3 nM), consistent with MVC-Res gp120 having a reduced affinity for CCR5. Similar results were obtained using ^35^S-MVC-Sens gp120 as a tracer (Figure 5g). Similarly to MVC-Res gp120, the MVC-Sens(V3R) variant gp120 showed weak ability to compete with ^35^S-MVC-Sens gp120 for binding to CCR5 (Figure 5g). On the contrary, removing Ala from the V3 loop of MVC-Res gp120 (Figure 5f), as well as substituting the V3 loop within MVC-Res gp120 by that of MVC-Sens gp120 (Figure 5g), resulted in an ability of gp120 to displace ^35^S-gp120 binding similar to that of MVC-Sens gp120.

Considered altogether, these results indicate that the MVC resistance-associated changes in the MVC-Res Env sequence, while increasing replication, impair the interactions with CD4 and CCR5, which could not be anticipated when using the 293-Affinofile receptor affinity profiling system.

### MVC-Sens and MVC-Res have different abilities to fuse with CD4^+^ T cells

We next investigated whether the different levels of replication between MVC-Sens, MVC-Res and their derived mutants are due to different entry efficiencies and/or kinetic rates. At this stage of the study, we also intended to assess whether the receptor binding properties of monomeric gp120 recapitulate those of the trimeric Env complex on the virus surface. Indeed, several previous works have suggested that the trimeric arrangement of Env may alter its binding to HIV entry receptors. For instance, recent studies have described that interactions between variable loops of adjacent gp120 subunits as well as gp120/gp41 interactions in the Env trimer are likely to modulate the degree of accessibility of the CD4-binding site [52–54]. It has also been reported that gp41 can regulate the resistance of HIV to low molecular weight CCR5 antagonists [25], presumably as a result of altered CCR5 usage.

To measure the ability of viruses to engage receptors and to enter into cells, we developed a virion-based fusion assay whose features have previously been described [55]. In this assay, we incorporated β-lactamase (BlaM)-vpr chimeric proteins into NL4-3Ren-derived viral clones expressing either of the MVC-Sens or MVC-Res gp160 variants (wt or mutants) and then measured the transfer of these chimeric proteins into the cytoplasm of activated CD4^+^ T-lymphocytes as a result of virus fusion. This transfer was detected by the enzymatic cleavage of the BlaM substrate CCF2-AM loaded in the target cells, resulting in the change of the CCF2 fluorescence emission spectrum from green (520 nm) to blue (447 nm). The number of CD4^+^ T-lymphocytes displaying cleaved CCF2 fluorescence was then quantified by flow cytometry (Additional file 2: Figure S2).

**Figure 6:**
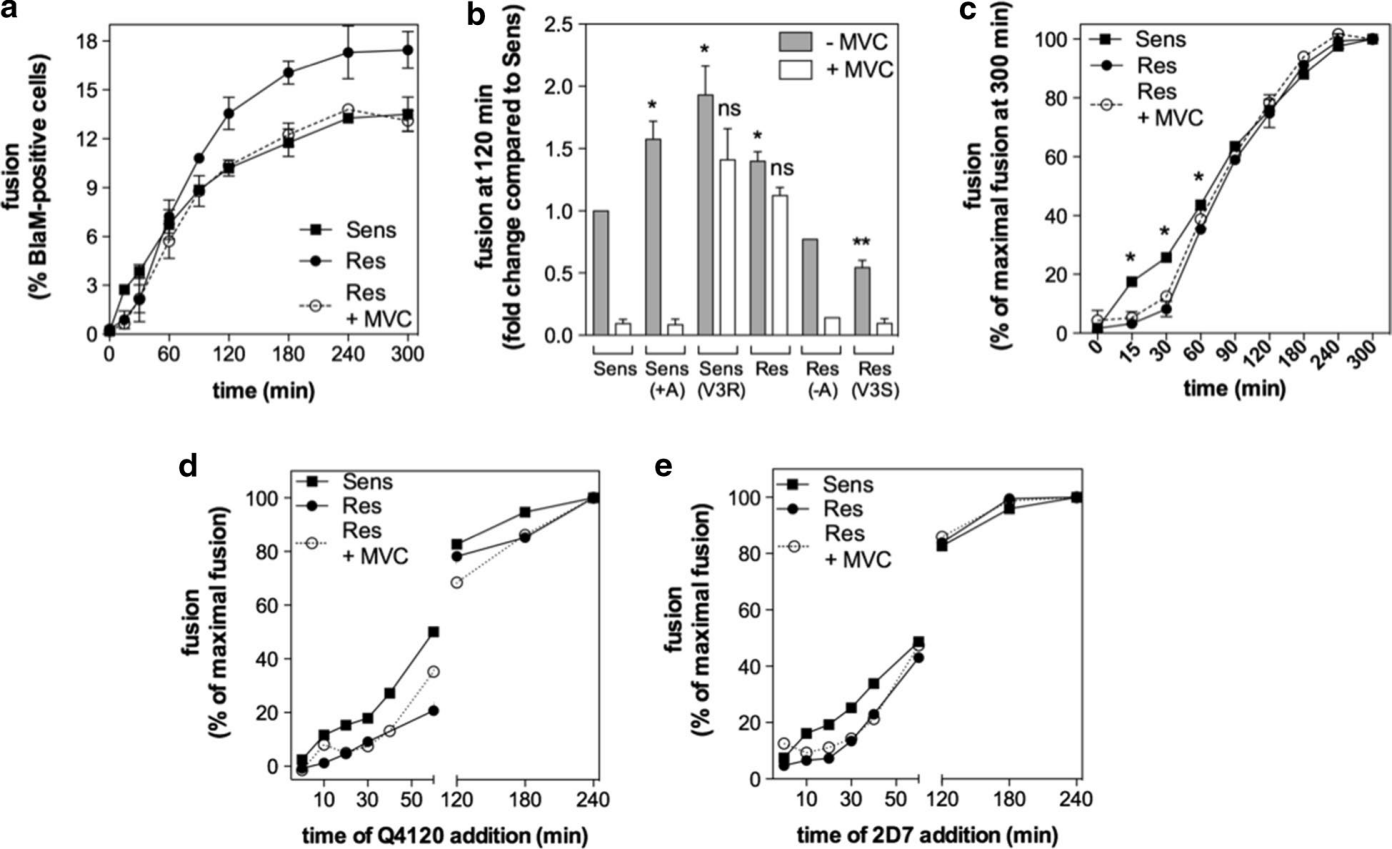
Characteristics of MVC-Sens and MVC-Res fusion with CD4^+^ T cells. **a** Fusion kinetics of BlaM-vpr-containing MVC-Sens and MVC-Res viruses with activated CD4^+^ T-lymphocytes are shown, in the presence or in the absence of 10 μM MVC. After virus spinoculation onto cells at 4°C and cell washing, fusions were run for the indicated times at 37°C and cells were then loaded with CCF2/AM. Results are expressed as the percentage of BlaM-vpr positive cells, *i.e.* cells displaying cleaved CCF2/AM fluorescence (at 447 nm). Results are mean ± SEM of two independent determinations out of at least five. **b** Levels of fusion at 2 h. for the viruses MVC-Sens, MVC-Res and their related variants Sens(+A), Sens(V3R), Res(−A) and Res(V3S), in the absence or in the presence of 10 μM MVC. Results, expressed as fold changes compared to the extent of fusion of MVC-Sens, represent mean ± SEM of 2–10 independent determinations. **c** The *panel* represents data from the fusion kinetics of MVC-Sens and MVC-Res w/or w/o 10 μM MVC that were normalized to the maximal extent of fusion at 300 min. **d** and **e** Time-of-inhibitor-addition experiments revealing that MVC-Res interacts with CD4 and CCR5 receptors more slowly than MVC-Sens. Fusion of viruses with CD4^+^ T cells was measured at 240 min under conditions where 50 μg/ml of the anti-CD4 mAb Q4120 (**d**) or 20 μg/ml of the anti-CCR5 mAb 2D7 (**e**) was added at the indicated time points after cell transfer to 37°C (time zero), in the presence or in the absence of 10 μM MVC. Results are expressed as the percentage of fusion relative to fusion in the absence of inhibitor (time 240 min). Representative experiments out of three independent experiments are shown.

Equal amounts (30–50 ng of Gag p24) of MVC-Sens and MVC-Res were first forced to attach to CD4^+^ T-lymphocytes by spinoculation for 60 min at 4°C, then cells were washed twice with culture medium and incubated at 37°C for different periods of time (Figure 6a, c). The extents of fusion (i.e. the amounts of cells expressing cleaved CCF2) increased over time and then reached a plateau value at 180–240 min that was ≈1.5-fold higher for MVC-Res than for MVC-Sens, consistent with MVC-Res fusing more efficiently with CD4^+^ T cells than MVC-Sens. The addition of 10 μM MVC decreased the maximal fusion of MVC-Res to a level that was not significantly different from that of MVC-Sens (Figure 6a, b; Additional file 2: Figure S2). In the same way as for viral replication (Figure 3b, d), we found that the V3 loop of MVC-Res, and more especially the insertion of Alanine, contributes to increasing the final extent of fusion (Figure 6b). Indeed, the MVC-Res(V3S) and MVC-Res(−A) variants lacking Alanine fused ≈2- to 3-fold less efficiently than MVC-Res. In contrast, inserting Alanine (MVC-Sens(+A)] or the entire MVC-Res V3 loop [MVC-Sens(V3R)] into the MVC-Sens sequence increased fusion to levels equal or even higher than that of MVC-Res. Overall, these results strongly suggest that the Ala-dependent increased replication of MVC-Res relates to its enhanced entry efficiency, which is counterbalanced to some extent in the presence of MVC.

After normalizing the kinetics to the final extents of fusion, it was apparent that the fusion of MVC-Res occurs at a slower rate at the early time points (up to 60 min), as compared to MVC-Sens, while both viruses fused at the same rate afterwards (Figure 6c). Of note, adding MVC, although decreasing the final extent of fusion of MVC-Res (Figure 6a), did not modify its rate of fusion, in particular at the early time points (Figure 6c). Previous experiments showed that viral fusion to target cells most often occur after a lag time, which represents the time needed for the virus to engage CD4 and CCR5, and in doing so to form what is named the ternary complex [10, 56]. The delayed fusion of MVC-Res might thus be related to the fact that the virus takes a longer time to engage CD4 and CCR5, compared to MVC-Sens, consistent with the lower affinity of MVC-Res gp120 for both receptors (Figure 5). Alternatively, other stages in the fusion of MVC-Res occurring after receptor binding might be slowed down. To discriminate between these two possibilities, we developed time-of-inhibitor-addition experiments, in which the extents of fusion of MVC-Sens or MVC-Res w/or w/o MVC were measured at 240 min under conditions where saturating concentrations of the anti-CD4 and anti-CCR5 mAbs Q4120 or 2D7 (50 and 20 μg/ml, respectively) were added at different times after the temperature rise to 37°C. Of note, these mAbs inhibit HIV attachment to target cells. The binding of 2D7 occurs at the N-terminal part of CCR5 ECL2, to which gp120 also binds, and is unaffected by MVC [14, 18]. Thus, we reasoned that if MVC-Sens and MVC-Res engage both receptors with similar kinetic rates, then they should be equally sensitive to inhibition by the mAbs. Alternatively, a greater sensitivity of either of the two viruses to mAbs would indicate that it engages receptors with a slower kinetics. In the representative experiments shown in Figure 6d, e, MVC-Res appeared to be more efficiently inhibited by both mAbs than MVC-Sens, especially at the early time points of fusion, indicating that the MVC-resistant virus has delayed kinetics of binding to CD4 and CCR5. This is consistent with our results in Figure 5 showing that MVC-Res gp120 has a lower affinity for both receptors, as compared to MVC-Sens gp120. In other words, these results strongly suggest that MVC-Res gp120 both in its monomeric form and as part of the trimeric Env complex on the virus surface is defective in its ability to bind to CD4 and CCR5. Interestingly, MVC did not affect the sensitivity of MVC-Res to 2D7 (Figure 6e), suggesting that MVC does not significantly modify the virus affinity for CCR5, and thus probably its capacity to interact with the CCR5 ECL2. Of note, the curves from the fusion kinetics (Figure 6c) and those derived from the time-of-inhibitor-addition experiments had similar overall shapes (Figure 6d, e), suggesting that the slower engagement of receptors mainly accounts for the delayed fusion of MVC-Res, while the other steps occurring after binding to CCR5 likely contributing little to this kinetic lag.

### 3-D modeling of the MVC-Sens and MVC-Res V3 loop structures and dynamics

**Figure 7:**
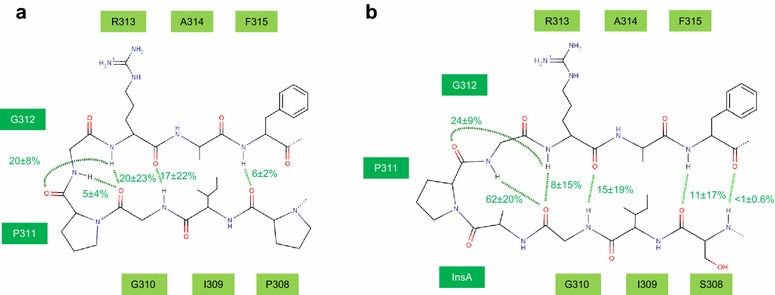
Organization of the MVC-Sens and MVC-Res V3 tips. The frequency of occurrence of hydrogen bonds in the V3 tips of MVC-Sens (**a**) and MVC-Res (**b**) Envs were calculated for every 25 ps segment of each molecular dynamics trajectory. The average rates and standard deviations calculated on the five trajectories are indicated near each H-bond noted with *dashed green lines.*

To better understand how the V3 loop changes in MVC-Res influence binding to CCR5 and resistance to MVC, we first ran molecular dynamics simulations of V3 loops from the MVC-Sens or MVC-Res isolates in explicit water. Our initial structures were modeled from the crystal coordinates of V3 in the context of the HIV-1 gp120 core complexed to CD4 and to the X5 antibody [3]. Five independent simulations of 100 ns each revealed that all the residues in the loops experience significant conformational fluctuations over time, even in the tip region (see supplemental text in Additional file 3 and Additional file 4: Figure S3 for details). The tip of the MVC-Res V3 loop appeared however slightly less flexible than that of MVC-Sens (Figures b and c in Additional file 4: Figure S3). Previous structural studies using NMR or X-ray crystallography techniques showed that the tip of V3 adopts a β-hairpin-like structure, with the GPG motif constituting the turn between the two anti-parallel strands [3, 9, 52]. Both MVC-Sens and MVC-Res V3 loops have an overall similar configuration, with a turn centered on P311 and three H-bonds between the upstream peptide (from P/S308 to G310) and the downstream peptide (from R313 to F315) in the tip (Figure 7). The presence of Ala in MVC-Res, however, induces the formation of a bulge in the turn, which alters the local conformation of the tip. This is notably manifested by an additional H-bond between G310 and G312 that was present in 62% of the structures simulated for the MVC-Res V3 loop (vs only 5% in MVC-Sens).

### Docking of the MVC-Sens and MVC-Res V3 loops into CCR5

**Figure 8:**
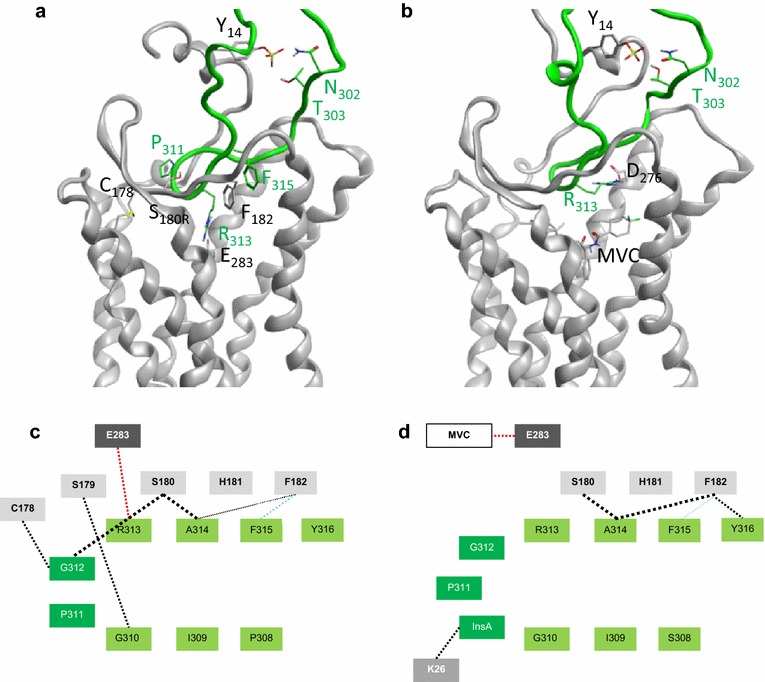
Molecular models of CCR5 binding the V3 tip of MVC-Sens Env (*left*) or the V3 tip of MVC-Res Env and MVC (*right*). **a** and **b** Three-dimensional view of the complexes. The backbone of CCR5 is represented as *grey ribbons* and the backbone of V3 as *green ribbons*. The side chains of amino acids involved in inter-molecular interactions are represented as *sticks*. MVC is represented as *sticks*. **c** and **d** Schematic view of binding modes. *Light, medium and dark grey boxes* represent the receptor residues in ECL2, N-terminus, and TM7, respectively. H-bonds are indicated with *dotted black lines*, aromatic stacking with a *dotted blue line* and ionic bonds with *dotted red lines*. The *thicker* the line, the more stable is the interaction.

We next constructed models of complexes formed between CCR5 and either of the two V3 loops (Figure 8). Docking of MVC-Res V3 was realized onto the MVC-bound CCR5 X-ray structure recently released [15] (Figure 8b). MVC-Sens V3 was docked onto the same CCR5 structure, however after removing the inhibitor (Figure 8a). The placement of V3 loops was guided by restraints derived from experimental data (in particular, we learned from the comprehensive collection of experimental data presented in Ref. [57]), and performed in such a way allowing the tip and the base of V3 to interact with ECL2 and the N-terminus domain of the receptor, respectively. Similarly to what we previously reported for Bx08 gp120 [13, 58], we found here that MVC-Sens V3 binds to an outer region of CCR5 that is distinct from the deeply buried binding site for MVC. In such a configuration, R313 in the tip of MVC-Sens (Figure 8a) and Bx08 establishes an ionic bond with E283 in CCR5 (Figure 8c), providing a structural explanation for the involvement of this acidic residue in high-affinity binding of the viral envelope glycoprotein [13]. Of interest, E283 is the only residue in CCR5 that is mandatory for MVC binding [13], making it possible that a competition takes place between MVC and gp120 for strong interaction with E283. Focusing on intermolecular interactions, the modeling of the MVC-Sens V3-CCR5 complex also predicted that a series of hydrogen bonds and aromatic interactions tightly pair the G310-F315 sequence in V3 and the G178-F182 sequence in the receptor ECL2 (Figure 8c). Although MVC-Sens and MVC-Res V3 loops roughly span the same domains of CCR5, MVC-Res V3 showed a different mode of CCR5 recognition (Figure 8b, d). Overall, it appeared that MVC-Res V3 stands slightly above than MVC-Sens V3 in CCR5. Also, MVC-Res V3 established a smaller amount of interactions with ECL2 as compared to MVC-Sens, hence providing a molecular basis for the decreased affinity of MVC-Res gp120 for CCR5. Interestingly, binding of the MVC-Res V3 tip to MVC-bound CCR5 was independent from ionic bonding to E283. The positive charge of R313 could nevertheless engage an ionic bond with D276, which is located in an upper part of the transmembrane cavity. Of note, an ionic bond between D276 and R313 was also observed during one-third of the simulation time for the complex between MVC-Sens and CCR5, suggesting that this interaction may help position the V3 tip but probably plays a minor role in locking it onto CCR5 (Additional file 5: Table S1). Modeling of the 3D-complex between CCR5 and MVC-Res V3, in the absence of MVC, confirmed that the inability to establish an extensive binding to ECL2 is an inherent property of MVC-Res Env.

## Discussion

Understanding how HIV-1 adjusts the use of entry receptors to escape drug inhibition and spread in patients is of prime clinical importance. From a therapeutic point of view, such knowledge should prove useful in the development of more efficient inhibitors that are less prone to generate resistant variants. In this regard, the recent development of the 293-Affinofile receptor affinity profiling system has provided valuable information on the relative efficiencies of CD4 and CCR5 usage by HIV-1 variants resistant to CCR5 allosteric inhibitors [16–18, 32, 39]. In particular, efficient usage of CCR5 by resistant viruses as revealed by low sensitivity vector angles θ was shown to be associated with high degrees of resistance (low MPI values), leading to the hypothesis that the degree of resistance is related to the virus ability to bind to inhibitor-bound CCR5 [17, 32]. However, this hypothesis does not take into account that the virus might also take advantage of other CCR5-dependent functions to improve viral entry and drug resistance. The MVC-resistant HIV-1 clinical isolate MVC-Res we studied here has similarities with other patient-derived resistant viruses [18, 32], in that it has a high level of resistance (Figures 2, 3) associated with high efficiency of free CCR5 usage in Affinofile cells (Figure 4), in agreement with previous observations from Gorry’s Laboratory [17]. But we showed here that it also displays a slower kinetics of CCR5 engagement than its MVC-sensitive counterpart (Figure 6), probably as a result of a reduced gp120 affinity for the coreceptor (Figure 5). This indicates that parameters other than affinity for CCR5 may contribute to the extent to which a virus appears to use CCR5 efficiently in the Affinofile system. Furthermore, the data with the Affinofile cells are consistent with MVC-Res using MVC-bound CCR5 slightly less efficiently than free CCR5 (Figure 4 and Ref. [17]), but this is unlikely related to a reduced ability of the virus to bind to MVC-bound CCR5, as the inhibitor does not modify the kinetics of CCR5 engagement by MVC-Res (Figure 6c, e).

The most striking result in the present study is related to the fact that MVC-Res retains high levels of fusion and replication that are higher than those of MVC-Sens in spite of a dramatically reduced ability to interact with CCR5. More generally, correlations exist between viral fitness and CCR5 binding affinities [59, 60]. However, previous results also showed that anti-CCR5 antibodies could strongly inhibit gp120 binding to the coreceptor while moderately affecting viral entry [8, 35], indicating that those processes could require different molecular determinants in CCR5. Our results are actually reminiscent of those of a previous work, showing that monomeric gp120 derived from clones of a HIV isolate resistant to the small-molecule CCR5 inhibitor AD101 failed to bind to CCR5-expressing cells, in contrast to gp120 derived from AD101-sensitive clones [22], suggesting that impairment of CCR5 inhibitor-resistant viruses to bind CCR5 is more common than previously expected. Previous studies reported that adaptive mutations in HIV-1 Env causing increased affinity for CD4 [61] or accelerating the fusion process [62] may compensate for impaired interaction with the coreceptor, but we showed here that MVC-Res has a three-fold lower affinity for CD4 (Figure 5a) and does not fuse more rapidly than MVC-Sens (Figure 6).

Where then would the increased replicative capacity of MVC-Res have come from? We show here that the Ala insertion between G310 and P311 in the V3 loop of MVC-Res attenuates the ability of the virus to bind to CCR5 and concomitantly increases its fusion (Figure 6b) and replication (Figures 2, 3) efficiencies. Control experiments showed that Ala does not alter the level of Env expression on the virus surface (Additional file 6: Figure S4). However, this positive effect of Ala on replication is no longer apparent in cells expressing high levels of CCR5 (Figure 3f), suggesting that Ala plays a role in allowing MVC-Res to utilize low levels of CCR5 for infection. This supports a role for this amino acid in allowing MVC-Res to use CCR5 efficiently, in accordance with our results using the 293 Affinofile cell system. It is increasingly appreciated that CCR5 exists in different conformations, which are unevenly distributed at the cell surface [46, 49, 50, 63]. In this context, the Ala insertion might change the MVC-Res gp120 conformation in such a way that the virus interacts preferentially with CCR5 forms that are colocalized with CD4 and/or are enriched in particular membrane domains where the coreceptor density is high. This hypothesis is actually in line with recent data showing that CCR5 inhibitor-sensitive and -resistant viruses recognize distinct conformations of the coreceptor [25, 49, 64]. In particular, a virus resistant to the small-molecule CCR5 inhibitor vicriviroc (VVC) interacting preferentially with CCR5 molecules localized in cholesterol-rich membrane domains (lipids rafts) has been reported [49]. The privileged recognition by MVC-Res of membrane regions at the cell surface where the CCR5/CD4 ratio is high would explain how the virus maintains a high efficiency of CCR5 usage in affinofile cells (Figure 4) while having a reduced affinity for CCR5. Alternatively, it is known that alterations in the lateral distribution of membrane receptors can occur as a consequence of ligand-induced conformational changes [65, 66]. Similarly, it could be that the binding of MVC-Res stabilizes a different CCR5 conformation, as compared to MVC-Sens, which might redistribute into discrete membrane regions. This particular CCR5 conformation could also induce distinct signaling pathways, in particular those involved in cytoskeleton rearrangements, which have been shown to mediate enrichment of receptors at the fusion site [67] and to play a role in the formation and expansion of the fusion pore [68].

Interestingly, MVC counteracts the positive effect Ala has on fusion (Figure 6a) and replication (Figure 4b, c) and more generally renders MVC-Res more dependent on higher CCR5 expression levels in 293 Affinofile cells without affecting the CCR5 binding affinity. Recent computational methods for the prediction of CCR5 conformational ensembles showed that MVC stabilizes a different set of receptor conformations, as compared to free CCR5 [69]. These MVC-bound CCR5 conformations could differ in their organization at the plasma membrane, in their ability to interact with CD4 and/or to induce signaling pathways useful for fusion, thereby leading to increasing the degree of CCR5 dependence of MVC-Res.

Here we identified that two types of sequence motifs in MVC-Res gp160 contribute to MVC resistance, although it is not clear whether they act in concert or separately. Firstly, we found that both the P308S mutation and the Ala insertion in the V3 loop of MVC-Res (but not either of these changes) are necessary to confer a high level of MVC resistance (Figure 3). Our docking studies predicted that due to the Ala insertion, the MVC-Res V3 tip does no longer compete with MVC for interaction with E283 in MVC-bound CCR5, in contrast to MVC-Sens and Bx08 Envs (Figure 8). It is, however, unlikely that this process contributes much to MVC resistance, because we observed that the presence of Ala alone in the context of MVC-Sens or MVC-Res Envs is not sufficient to increase resistance to the inhibitor. Whether amino acids outside V3 modulate the extent of MVC resistance of MVC-Res, as shown in other Env contexts [18, 19], is, however, not known. Secondly, regions outside of the V3 loop in MVC-Res Env are sufficient to confer partial resistance to MVC on PBMCs (Figure 3c) and HEK cells expressing high levels of CCR5 (Figure 3e). This was revealed by incomplete inhibition of MVC-Res-derived mutants of the V3 loop by 10 μM MVC. The MVC-Sens isolate also partly resisted inhibition by MVC in HEK cells, albeit substantially less efficiently than the MVC-Res mutants, but in this case, we believe that this is more likely related to increases in the MVC IC_50_ due to high receptor expression levels, as previously reported for other MVC-sensitive viruses [14, 40], rather than to a genuine basal resistance to the drug. Previous work showed that resistance of R5 viruses to MVC in MVC-treated patients could occur with no changes in the V3 loop [28]. In the case of MVC-Res however, it is uncertain whether sequence changes outside V3 conferring MVC resistance arose as a consequence of a MVC selection pressure. Indeed, recent data showed that partial resistance to MVC at baseline is a common property shared by one-half of clade B and C R5 viruses from the chronic stage of infection [38]. In this context, it could be that MVC-Res derives from a minor quasispecies variant coexisting with MVC-Sens and already resistant to MVC before treatment, rather than from MVC-Sens itself. This variant could then become dominant thanks to the sequence changes in V3 (especially the Ala insertion) during MVC treatment. This hypothesis is actually consistent with what has been shown for the CC1/85 strain, which shows an inherent, low level of resistance to CCR5 allosteric inhibitors, which is predisposed to be dramatically increased under the selective pressure of these inhibitors [40].

Finally, analyzing the phenotypic properties of MVC-Res in this study pointed to important mechanisms for HIV-1 binding to CD4 and CCR5. We observed that MVC-Res has a threefold lower affinity for CD4, as compared to MVC-Sens, and that this was not related to the changes in the V3 loop (Figure 5a). Among the sequence elements that could account for this result, significant changes in the V1/V2 loops of MVC-Res (see Figure 1) are likely to affect the degree of aperture of the CD4 binding site [45]. Regarding binding to CCR5, our results showing that MVC-Sens and MVC-Res gp120 have similar affinities for the mAbs 17b and E51, together with recent molecular modeling studies [17], suggest that the sequence elements of MVC-Res gp120 allowing binding to the CCR5 N-terminus (the bridging sheet and the base of V3) are preserved. However, our results also strongly suggest that those interactions are not sufficient to maintain a high affinity for CCR5 and support a model wherein engagement of HIV gp120 at both the CCR5 N-terminus and ECL2 is necessary for conferring strong binding to the coreceptor. Indeed, we provide here sound evidence that MVC-Res Env has a reduced affinity for CCR5, and our molecular dynamics simulations and modeling suggest that this could be due to less extensive interactions between the V3 tip and CCR5 ECL2. Interestingly, loose interaction of MVC-Res gp120 with CCR5 ECL2 occurred regardless of the presence of MVC (Figure 8), thereby reinforcing our conclusion from the experimental data that MVC has no influence on the already low MVC-Res gp120 affinity for CCR5. One can assume however that a link exists between disrupted interactions with ECL2 and the occurrence of resistance to MVC, as suggested in previous work where deletion of V3 has been shown to result in viruses being fully resistant to CCR5 allosteric inhibitors [70–72]. As mentioned above, it has been described that one-half of clade B and C HIV-1 isolates using CCR5 as a coreceptor exhibits intrinsic resistance to MVC, but no clear phenotypic or genotypic signatures have been identified [38]. It remains an open question if those viruses share the property to have succeeded in growing while freeing themselves from the obligation to establish strong interactions with ECL2.

## Conclusions

Virological failures in patients on MVC therapy are documented but the knowledge on how R5 HIV-1 strains adjust the use of entry receptors to resist MVC and to propagate in patients, although of clinical importance, is still incomplete. Here, we combined virological and pharmacological approaches with molecular dynamics simulations to demonstrate that amino acid changes in the viral envelope glycoprotein (Env) associated with MVC-resistance can lead to reduced affinities for CD4 and CCR5 while maintaining high efficiency of viral entry and replication. Among the determinants causing MVC resistance, we identified in a MVC-resistant virus, which had emerged as a dominant viral quasispecies in a patient, that a single amino acid insertion in the Env V3 loop decreases CCR5-binding affinity, but, at the same time, augments viral entry. Additional data in cells with varying CCR5 levels also suggested that the insertion increases the efficiency of CCR5-dependent steps of viral entry other that Env attachment. Overall, these results shed light on a new route through which MVC-resistant viruses could emerge and grow in treated patients.

## Methods

### Ethics statement

Blood samples from healthy donors were obtained from Etablissement Français du Sang (EFS, the French National Blood Agency). Sample use for scientific aim has been approved by the French Research Ministry under the code: DC-2008-68 Coll 2 “EFS” and by the French Ethical Committee “CPP Ile de France I” Including College I and College II (President Dr Elisabeth Frija) on April 30th 2009 under the code 08-11887. Consent of donors was obtained according to the EFS procedure, which has been approved by the competent authorities. All samples were coded as required by the French privacy agency (so called CNIL, Commission Nationale de l’Informatique et des Libertés) (CNIL law: 78-17-January 6th 1978-modified). The only biological data available with these samples are the results of systematic serological screening tests: anti HIV-1 and HIV-2, anti-HCV and HBsAg.

### Cell culture and reagents

The procedures for the purification of human peripheral blood mononuclear cells (PBMCs) and CD4^+^ T-lymphocytes were previously described [14, 46]. Cells were maintained for 2 days in RPMI 1640 medium containing IL-2 (300 IU/ml) and phytohemagglutinin (5 μg/ml), and then for additional 4–6 days in the presence of IL-2 alone. CCR5-expressing HEK 293T cells and the U87-CD4/CCR5 cell line were previously described [14, 46]. The transient expression of CD4 in CCR5-expressing HEK 293T cells was carried out as described [14]. HEK 293T cells stably expressing CD4 (HEK-CD4 cells) were generated by transduction with the TRIP ΔU3 lentiviral vector (a gift from Dr P. Charneau, IP, Paris) encoding the receptor sequence. The 293-Affinofile cell line (a gift from Dr B. Lee, Mont Sinai Hospital, New York, NY, USA) was maintained in DMEM supplemented with 10% (v/v) foetal bovine serum, 100 μg/ml streptomycin, 100 units/ml penicillin, and 50 μg/ml blasticidin (Invitrogen). Recombinant soluble human CD4 (sCD4) was produced in the S2 cell line and purified on a strep-Tactin column using the One-STrEP-tag fused to the CD4 C-tail as a bait (Dr S. Pêtres, Plate-forme protéines recombinantes, Institut Pasteur). TAK779, MVC and the mAbs 17b and E51 were obtained from the AIDS Research and Reference Reagent Program catalog of the National Institutes of Health (Bethesda). The anti-CCR5 mAb 2D7 was obtained from BD Biosciences. The anti-CD4 mAb Q4120 was provided by Dr Q. Sattentau and the NIBSC Centralised Facility for AIDS Reagents.

### Viral clones

The sequences encoding the MVC-Sens and MVC-Res Envs were cloned into the pNL-KspI/Env/NotI-Ren vector derived from the HIV-1 proviral clone pNL4-3Ren [36] to produce replication-competent viruses expressing the Renilla Luciferase reporter gene. The KspI restriction site was introduced at the nucleotide position 6214 in pNL4-3Ren resulting in substitution of Ser for Arg-52 in the *vpu* gene. The Env genes were amplified by PCR using the cDNAs provided by Pfizer as templates and the forward (5′-TCCCCGCGGCAATGAGAGTGAAGGGGA-3′) and reverse (5′-ATAAGAATGCGGCCGCGCCACCCATCTTATAGCATAGC-3′) primers and then inserted between the KspI and NotI sites into the pNL-KspI/Env/NotI-Ren vector. DNA sequences of the cloned full-length Envs were confirmed by sequencing. The V3 loop of MVC-Res Env contains two changes (P308S and G310_P311InsAla) compared to MVC-Sens. Mutant clones introducing one or both of these V3 loop changes in MVC-Sens or reversing them from MVC-Res Env were created by site-directed mutagenesis. The pBx08Ren and pJRRen plasmids, which contain the gp160 from the Bx08 and JR-CSF HIV-1 strains, were described previously [14].

### Infection inhibition assays

The protocols for the preparation and titration of Renilla luciferase reporter viruses have previously been reported [14]. Drug susceptibility assays using PBMCs, U87-CD4/CCR5 and HEK-CD4/CCR5 cells as target cells were carried out as described [14], except that MVC and TAK779 were added to the cells 2 h prior virus inoculation (10 ng p24 Gag/well). Cells were incubated for 30 h (U87 and HEK) or 48 h (PBMCs) at 37°C before being lysed. Viral replication was then determined by measuring luciferase activity (Renilla Luciferase Assay, Promega, Madison, WI, USA) in the cell lysates using the 96-well plate luminometer “Orion” (Berthold). Background activity was assessed in the presence of 5 µM Zidovudine (AZT, ZDV) and was subtracted from all wells. The percentages of inhibition were calculated as [1 − (luciferase activity in the presence of drug/luciferase activity in the absence of drug)] × 100. Curve fitting and IC_50_ calculations were performed with the Prism Software using a sigmoidal dose–response model with a variable slope.

### Affinofile assay

The 293-Affinofile cells were seeded onto 96-well plates at a density of 5 × 10^4^ cells/well and then cultured for 24 h as described above. Thirty populations of cells expressing varying levels of CD4 and CCR5 were then generated by inducing cells with two-fold serial dilutions from 0.625 to 5 ng/ml of minocycline (Sigma) (CD4 induction) and from 0.125 to 2 μM of ponasterone A (Invitrogen). Cells were then incubated for 18 h at 37°C, after which receptor expression levels and virus infectivities were measured. Determining CD4 and CCR5 expression levels at the cell surface was performed by incubating cells at 4°C for 1 h with saturating concentrations of monoclonal antibodies (0.5 or 1 mg/ml) against either CCR5 (clone CTC5, R&D systems) or CD4 (clone OKT4, eBioscience) and then with a PE-labelled anti-mouse IgG antibody (BD Biosciences, ref #550589). Analysis of the PE fluorescence was carried out on a CANTO flow cytometer (BD Biosciences). In parallel, a conversion ratio between the measured mean fluorescence intensities of PE-labelled receptors and the amount of CCR5 molecules at the cell surface was determined by means of saturation experiments of ^125^I-CCL3 binding to CCR5-expressing HEK 293T cells, carried out as described previously [14]. This method allowed us to determine CD4 and CCR5 expression levels at the surface of 293-Affinofile cells that fell well within the range of those described in previous works (see the legend of Figure 4 and Ref. [34] for comparison). Regarding infections, the induced 293-Affinofile cells were also incubated or not with 10 μM MVC and then inoculated with the Renilla luciferase-expressing MVC-Sens or MVC-Res viruses (10 ng of Gag p24/well). The luciferase activity was determined 30 h post-infection as described above. The metrics describing the viral isolates’ infectivity features (see text for details) were then mathematically calculated using the VERSA computational platform (http://versa.biomath.ucla.edu), as described previously [34, 73].

### Production, ^35^S-labeling, and purification of soluble monomeric gp120

The gp120 coding sequences from the eight viral clones derived from MVC-Sens and MVC-Res Envs were amplified by PCR and then inserted into the previously described Semliki forest virus-derived expression vector pSFV2 [14]. Soluble, monomeric gp120 glycoproteins were produced and metabolically labeled with ^35^S-Cysteine and Methionine as described in Ref. [14]. They were then purified by affinity chromatography on Strep-Tactin columns (IBA) using the One-STrEP-tag fused to the gp120 C-terminus as a bait, and their concentrations were determined by Coomassie blue staining using BSA as a standard.

### Binding assays

Displacement experiments of Q4120 binding to HEK-CD4 cells by gp120s were performed as follows. Cells (5 × 10^5^) in 96-well conical bottom plates were incubated for 2 h at room temperature in 0.2 ml of binding buffer (50 mM HEPES, pH 7.4, 5 mM MgCl_2_, 1 mM CaCl_2_, 1% BSA, and 0.1% NaN_3_) containing 0.4 nM Q4120 and increasing concentrations of unlabeled gp120s. Cells were then centrifuged (211×*g* for 5 min at 4°C), incubated at 4°C for 1 h with Alexa Fluor 647-conjugated goat anti-mouse IgG (dilution 1:500) (Life Technologies) in 0.05 ml PBS supplemented with 1% BSA and 0.1% NaN_3_ and finally washed once in the same buffer. Non-specific binding was determined similarly using control IgG1 (BD Biosciences) as tracer or parental HEK 293T cells. Specific Q4120 binding to CD4 was then calculated by subtracting non-specific binding from total binding of the mAb, as measured by flow cytometry analysis (FACSCanto, BD Biosciences). The IC_50_ values for half-maximal inhibition of Q4120 binding by the gp120s was determined with the Prism Software using a one-site competitive binding model. The dissociation constants K_i_ for the gp120s were calculated according to the Cheng and Prusoff equation K_i_ = [IC_50_/(1 + L/K_D_)] [74], where L and K_D_ represent the Q4120 concentration and the dissociation constant of the Q4120-CD4 complex (K_D_ = 0.4 nM, see text), respectively. The binding experiments of ^35^S-gp120 to CD4 were performed in eppendorf tubes as follows. Crude membranes from HEK-CD4 cells (10 μg of proteins), which were prepared as described previously [75], were incubated for 2 h at room temperature in 0.1 ml of binding buffer containing 5% BSA and the radiolabeled glycoproteins used at a concentration equal to their K_i_ value for CD4. Non-specific binding was determined using the CD4-negative, parental HEK 293T cells. Bound and unbound ^35^S-gp120 were then separated by centrifugation (4°C, 5 min, 15,800×*g*) and removal of the supernatant. Membranes were then washed once with the washing buffer (50 mM HEPES, pH 7.4, 1 mM CaCl_2_, 5 mM MgCl_2_, 500 mM NaCl) and resuspended in Optiphase Supermix scintillation liquid (PerkinElmer Life Sciences). Bound radioactivity was measured in a Wallac 1450 Microbeta TriLux (PerkinElmer Life Sciences). Saturation binding experiments of ^35^S-gp120 to CCR5-expressing membranes were performed in eppendorf tubes in the presence of 400 nM sCD4, as previously described [14]. For the displacement experiments of 10 nM ^35^S-MVC-Sens gp120 or ^35^S-gp120_Bx08_ binding to CCR5-expressing membranes by unlabeled gp120s, thirty μg of membrane proteins were incubated in eppendorf tubes for 2 h at room temperature in 0.1 ml of binding buffer containing 5% BSA, the radioactive and unlabeled glycoproteins and 1 μM sCD4. Membranes were subsequently treated as the CD4-expressing membranes. Non-specific binding of ^35^S-gp120 to CCR5-expressing membranes was determined in the presence of 10 μM TAK779. For the analysis of the interactions between gp120s and anti CD4i antibodies by surface plasmon resonance experiments, *N*-ethyl-*N*′-(diethylaminopropyl)-carbodiimide/*N*-hydroxy-succimide activated CM4 sensorchips were functionalized with 2,300 or 1,800 RU of mAbs 17b or E51, respectively. Experiments were then carried out as described previously [47].

### HIV-1-CD4^+^ T cell fusion assays

The procedure for the production of BlaM-vpr containing viruses was described previously [76]. CD4^+^ T cells (1 × 10^5^) were inoculated with the BlaM-Vpr-containing viruses (50 ng p24 Gag), spinoculated for 1 h at 4°C, washed once with culture medium and then incubated for different times at 37°C. In time-of-inhibitor-addition experiments, fusion was measured at 240 min under conditions where 50 μg/ml of the anti-CD4 mAb Q4120 or 20 μg/ml of the anti-CCR5 mAb 2D7 was added at the indicated time points after cell transfer to 37°C (time zero), in the presence or in the absence of 10 μM MVC. Cells were then incubated with the CCF2/AM dye (at a 1.85 μM final concentration) for 2 h at room temperature in CO_2_-independent medium supplemented with 10% FBS. Cells were then washed with CO_2_-independent medium and then fixed in 2% paraformaldehyde. Enzymatic cleavage of CCF2/AM by BlaM, which results in a change of the CCF2 fluorescence emission spectrum from green to blue, was measured by flow cytometry (FACSCanto, BD Biosciences).

### Molecular dynamics simulations of gp120 V3 loops

Initial structures were modeled from the crystal coordinates of V3 in the context of the HIV-1 gp120 core complexed to CD4 and to the X5 antibody [3]. The residues of the gp120 V3 loop ranging from C296 to C331 were extracted from the PDB file 2B4C and edited using Sybyl-X 2.1.1 (Tripos Software Inc, El Cerrito, CA, USA). The alanine insertion in the 24-Res model was obtained by replacing the GPG segment with the GAPG segment using the protein loop search option of Sybyl. Using tleap in AMBER 12 (University of California, San Francisco, CA, USA), each loop was embedded in an octahedral box containing 5,150 water molecules and three Cl^−^ ions. The energy of the input system was minimized through 5,000 steps of steepest descent followed by 5,000 steps of conjugate gradient. The system was then equilibrated in a two-step protocol including harmonic constraints on backbone atoms. In the first step, the system was heated from 100 to 300 K using the Langevin dynamics algorithm during 20 ps. In the next step, harmonic constraints on backbone atoms were decreased from 1 to 0 kcal/mol.Å^2^ during 300 ps. Each equilibrated system was subjected to constant temperature (300 K) and pressure (1 atm) production run of molecular dynamics (MD) simulations. Five dynamics of 100 ns each were carried out for the MVC-Sens and the MVC-Res V3 loops. Each production run had a random initial velocity assignment. All MD simulations were performed using AMBER 12 with the leaprc.ff03.r1 force field [77]. The ptraj software from AmberTools package was used to analyze the MD trajectories.

### Docking of the gp120 V3 loops into CCR5

The crystal structure of CCR5 was prepared from the 4MBS PDB file [15]. The rubredoxin fusion protein used for crystallization was replaced by the third intracellular loop of the receptor. The missing N-terminus was added based on the NMR structure of the peptide S7-Y15 determined in complex with gp120 (PDB code 2RLL, [9]). Sulfate groups replaced the hydroxyl groups of Y10 and Y14. The V3 loop was manually docked into CCR5 under constraints as follows: (1) the C-terminus including the tip (residues 308–330) was modeled from the crystal coordinates of V3 in the context of the HIV-1 gp120 core complexed to CD4 and to the X5 antibody (PDB code 2B4C) [3] and positioned into CCR5 so as to reproduce the H-bonds established between CXCR4 ECL2 and the peptide CVX15 (PDB code 3OE0, [78]) and to bring the V3 R313 side chain close to the CCR5 E283 carboxylate; (2) the V3 N-terminus (residues 296–307) was modeled from the crystal coordinates of V3 in the context of the HIV-1 gp120 core complexed to CD4 and to the 412d antibody containing two sulfotyrosines (PDB code 2QAD, [9]) and positioned into CCR5 so as to bring the side chains of N302 and T303 close to CCR5 Y14. Modifications in V3 to match to MVC-Sens and MVC-Res sequences were performed as described above. Molecules were handled and edited using MOE 2013 (Chemical Computing Group, Montreal, QC, Canada). The complex was then placed into a hydrated lipid bilayer and relaxed using MD with AMBER 14 (University of California, San Francisco, CA, USA). In detail, 84 palmitoyloleoylphosphatidylcholine (POPC), 84 palmitoyloleoylphosphatidylethanolamine (POPE) and 42 cholesterols composed the bilayer that was surrounded by 15,877 water molecules, 55 Cl^−^ ions and 43 K^+^ ions. After energy minimization, the system was heated to 100 K at constant volume during 500 ps, while fixing the positions of all atoms except in water using harmonic constraints of 10 kcal/mol.Å^2^. In a second stage of 500 ps heating, the temperature was raised to 300 K at constant pressure, keeping only the CCR5 and V3 atoms rigid. The equilibrated system was then subjected to constant temperature (300 K) and pressure (1 atm) simulations. Distance and angular restraints between V3 and CCR5 and constraints on receptor atom positions were imposed during the simulation according to the schemes given in Additional files 7: Figure S5 and 8: Figure S6. The representative structures of the complexes were obtained by clustering the last 120 frames corresponding to 60 ns simulation without constraints or restraints between the V3 tip and CCR5. Intermolecular interactions between the V3 tip and CCR5 ECL2 monitored during the last 60 ns of simulation were reported in Additional file 5: Table S1.

## Additional files

Additional file 1:
**Figure S1.** Insertion of Ala in the GPG crown impairs the replicative capacity of the HIV-1 strains Bx08 and JR-CSF. U87-CD4/CCR5 cells were infected by 10 ng Gag p24 of the indicated viral isolates and luciferase activity in the cell lysates was measured 24 h post-infection. Results are expressed as percent replication of the Ala-containing viruses relative to that of their wild-type counterparts (100%). The RLU levels for Bx08 and JR-CSF were ≈ 900000 and 700000, respectively. A representative experiment out of three independent experiments performed in triplicate is shown. Additional file 2:
**Figure S2.** Effects of MVC on fusion of MVC-Sens or MVC-Res to CD4^+^ T-lymphocytes. BlaM-Vpr-containing viruses (50 ng p24 Gag) were incubated for 3 h. at 37°C with 1x10^5^ CCF2-loaded, activated CD4^+^ T-lymphocytes in the presence or in the absence of 10 μM MVC. Viral fusion was evaluated by measuring the enzymatic cleavage of CCF2 by flow cytometry. The panels indicate the percentages of cells positive for cleaved CCF2. A representative experiment is shown. Additional file 3: Supplemental text (related to Figure 7 and additional file 4). Additional file 4:
**Figure S3.** Structure and dynamics of free V3 from the MVC-Sens (grey) or MVC-Res (black) isolates. (A) Time series of root mean square deviation (RMSD) values for all atoms of the V3 loops, using as reference the average coordinates computed from the five independent simulations. (B) The phi and psi angular fluctuations were calculated and averaged by residue for every 25 ps segment of the five molecular dynamics trajectories. Standard deviations are reported for phi (top) and psi (bottom) backbone torsion angles. (C) The average RMS fluctuations (RMSF) were calculated for the carbon alpha atoms of the V3 loop residues from the ensemble of structures after superimposition of residues 296 to 330 (complete V3 loop, plain lines) or superimposition of residues 308 to 315 (V3 tip, dotted lines). Additional file 5:
**Table S1.** Intermolecular interactions between the V3 tips and CCR5. Additional file 6:
**Figure S4.** Western blot analysis of gp120 and Gag p24 expression on MVC-Sens, MVC-Res, MVC-Sens(V3R) and MVC-Res(V3S) viral particles. Three hundred ng Gag p24 of the different viral isolates were solubilized in lysis buffer (100 mM (NH_4_)_2_SO_4_, 20 mM Tris-HCl (pH 7.5), 10 % glycerol, and 1% Triton X-100) and then loaded onto Biorad Criterion^TM^ XT 4-12% Bis-Tris Gels under reducing conditions. Virus-associated gp120 and p24 were visualized using a sheep anti-HIV-1 gp120 polyclonal antibody (AALTO Bio Reagents LTD.) and a mouse anti-HIV-1 p24 monoclonal antibody (clone 183-H12-5C) (NIH AIDS Reagent Program), respectively, and then with the appropriate HRP-conjugated secondary antibodies (VECTOR). Bands were developed by enhanced chemiluminescence (Thermo Scientific) and were quantified using a LAS-1000 CCD camera (Image Gauge Software, Fuji Film Co., Tokyo, Japan). The figure shows that the viral isolates have gp120/p24 ratios R that are in the same range (R = 1, 1.15, 1.24 and 0.81 for MVC-Sens, MVC-Res, MVC-Sens(V3R) and MVC-Res(V3S), respectively). A representative experiment out of two is shown. Additional file 7:
**Figure S5.** Molecular modeling of the complex between CCR5 and MVC-Sens V3. The graphic shows time series of RMSD values of the complex using as reference the input coordinates. The table indicates which constraints or restraints were imposed on the system during the simulation. bb : backbone atoms, all : all atoms, sc : side chains. Additional file 8:
**Figure S6.** Molecular modeling of the complex between MVC-bound CCR5 and MVC-Res V3. The graphic shows time series of RMSD values of the complex using as reference the input coordinates. The table indicates which constraints or restraints were imposed on the system during the simulation. bb : backbone atoms, all : all atoms, sc : side chains.
