# Supplementary figures and images for: A single-residue change in the HIV-1 V3 loop associated with maraviroc resistance impairs CCR5 binding affinity while increasing replicative capacity
Source: Retrovirology. 2015 Jun 18;12:50. doi: 10.1186/s12977-015-0177-1 (PMC4470041; doi:10.1186/s12977-015-0177-1)

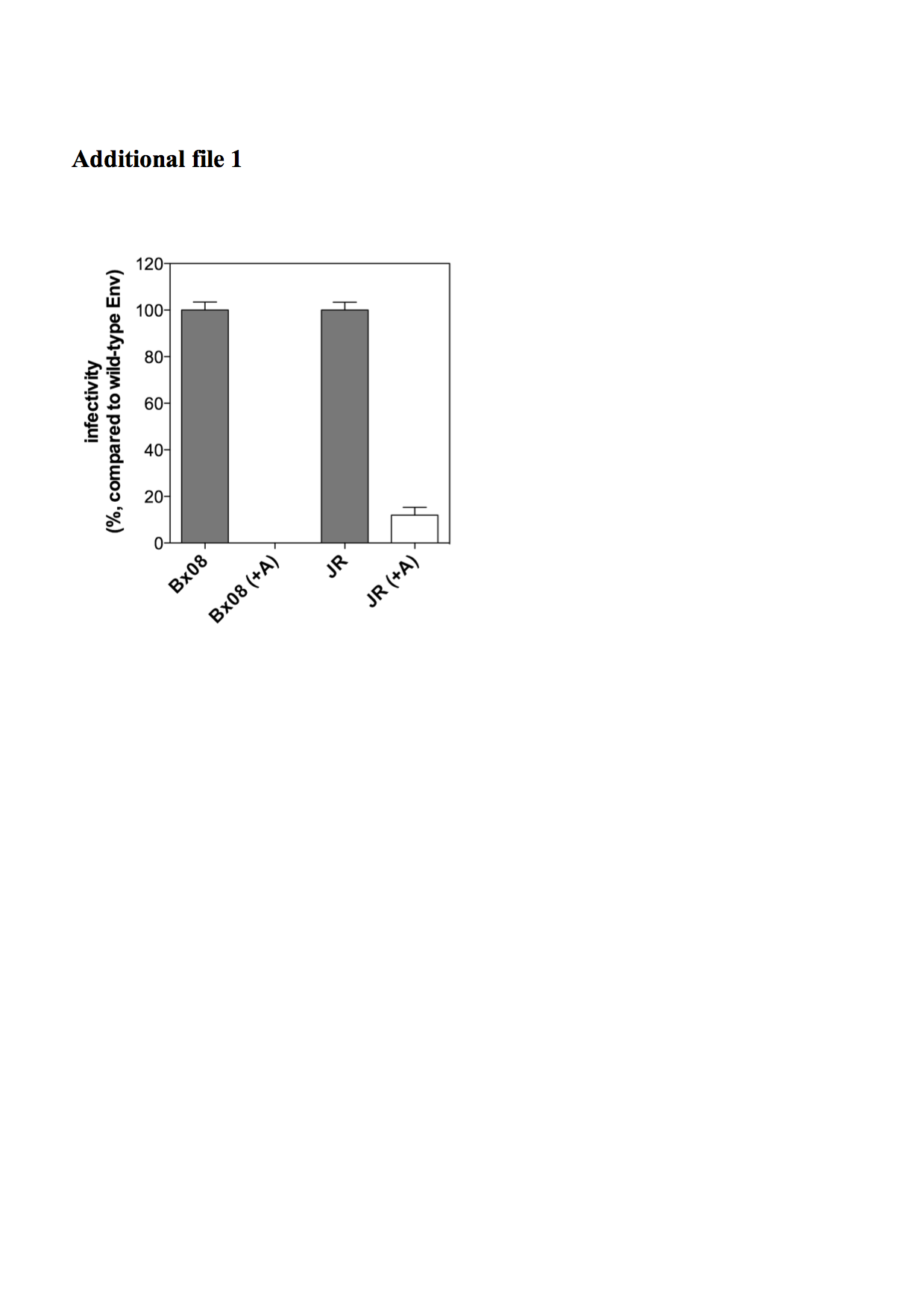

Supplement: Additional file 1: — Figure S1. Insertion of Ala in the GPG crown impairs the replicative capacity of the HIV-1 strains Bx08 and JR-CSF. U87-CD4/CCR5 cells were infected by 10 ng Gag p24 of the indicated viral isolates and luciferase activity in the cell lysates was measured 24 h post-infection. Results are expressed as percent replication of the Ala-containing viruses relative to that of their wild-type counterparts (100%). The RLU levels for Bx08 and JR-CSF were ≈ 900000 and 700000, respectively. A representative experiment out of three independent experiments performed in triplicate is shown. [file 12977_2015_177_MOESM1_ESM.tiff]

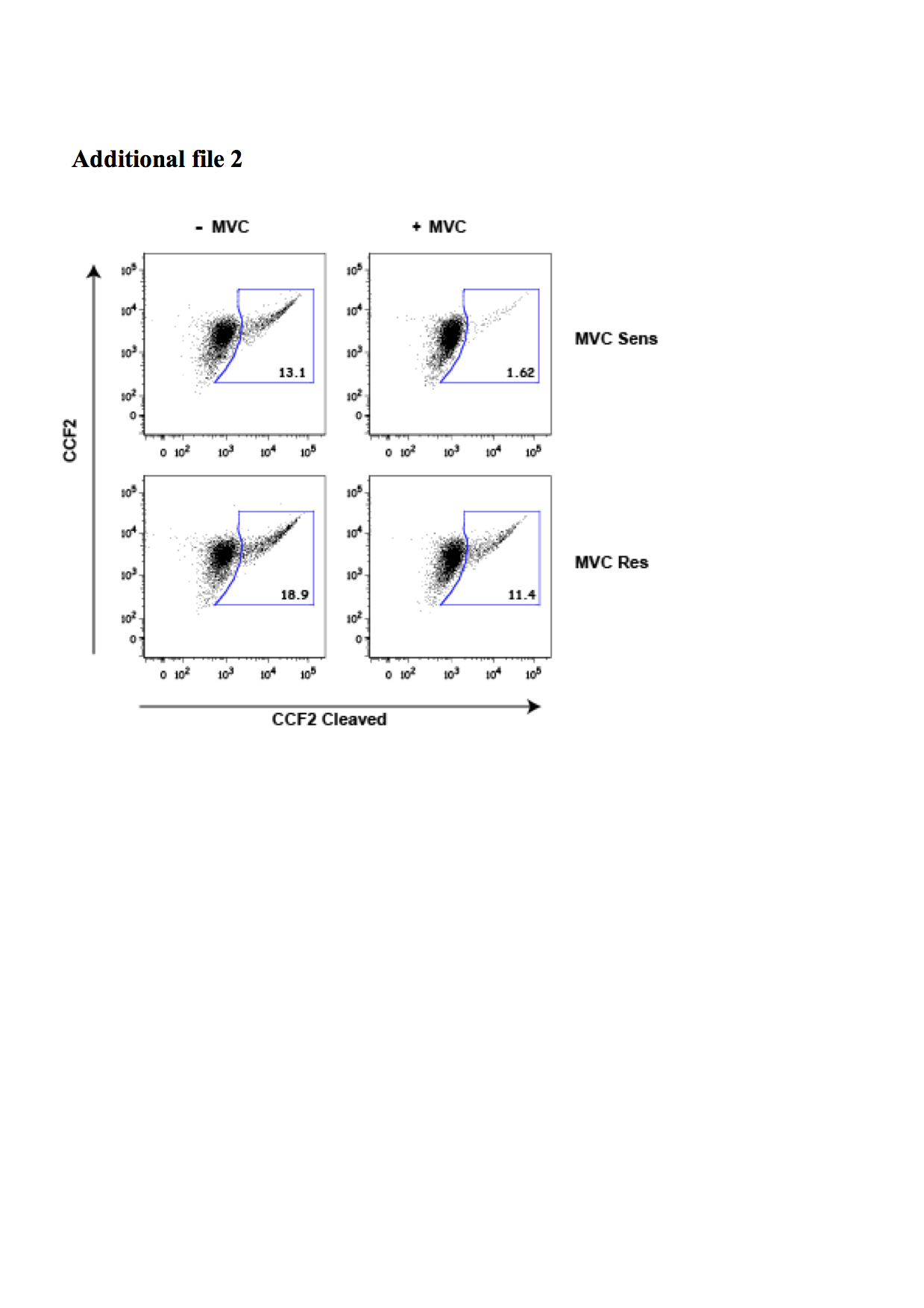

Supplement: Additional file 2: — Figure S2. Effects of MVC on fusion of MVC-Sens or MVC-Res to CD4+ T-lymphocytes. BlaM-Vpr-containing viruses (50 ng p24 Gag) were incubated for 3 h. at 37°C with 1x10^5^ CCF2-loaded, activated CD4+ T-lymphocytes in the presence or in the absence of 10 μM MVC. Viral fusion was evaluated by measuring the enzymatic cleavage of CCF2 by flow cytometry. The panels indicate the percentages of cells positive for cleaved CCF2. A representative experiment is shown. [file 12977_2015_177_MOESM2_ESM.tiff]

Additional file 4

a

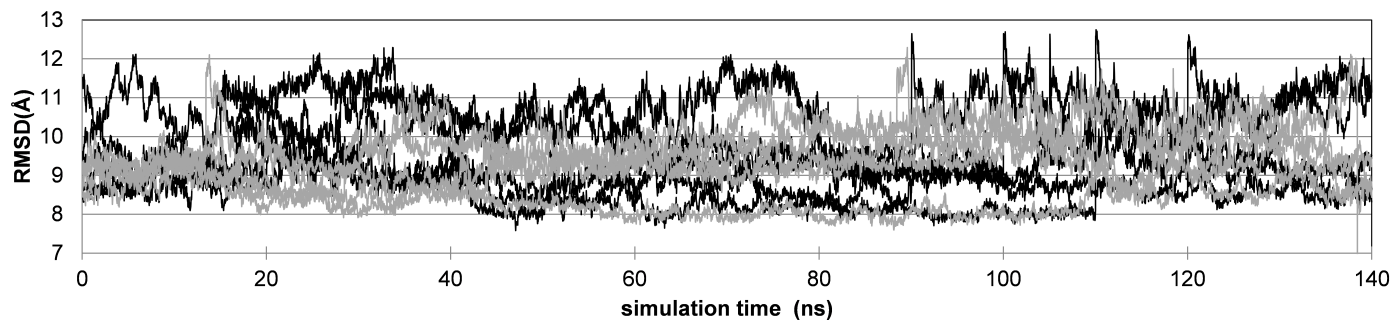

b

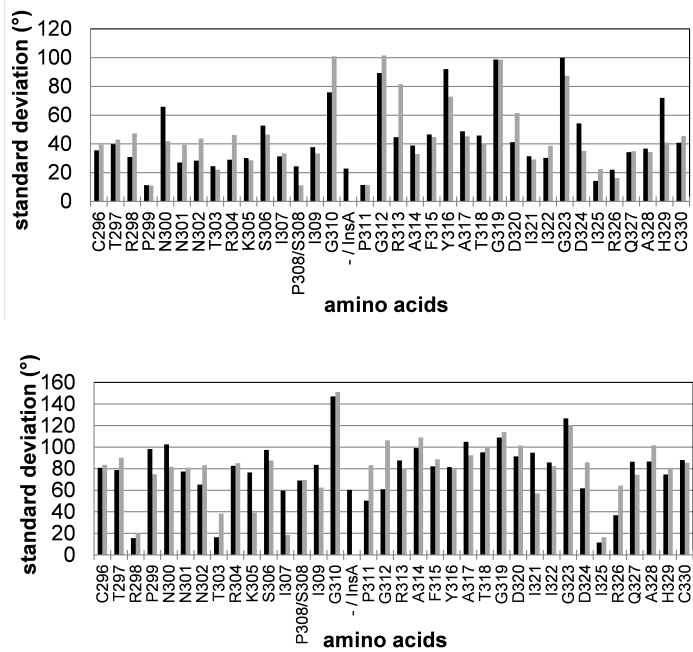

c

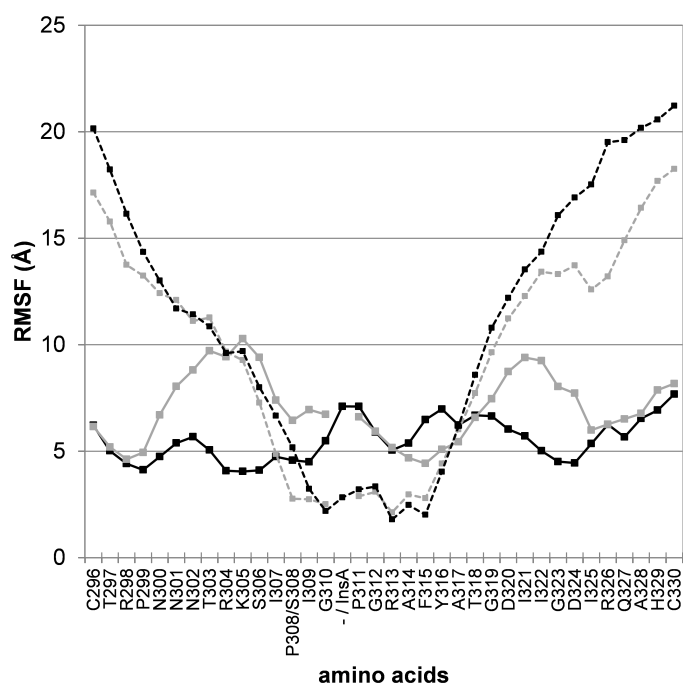

Supplement: Additional file 4: — Figure S3. Structure and dynamics of free V3 from the MVC-Sens (grey) or MVC-Res (black) isolates. (A) Time series of root mean square deviation (RMSD) values for all atoms of the V3 loops, using as reference the average coordinates computed from the five independent simulations. (B) The phi and psi angular fluctuations were calculated and averaged by residue for every 25 ps segment of the five molecular dynamics trajectories. Standard deviations are reported for phi (top) and psi (bottom) backbone torsion angles. (C) The average RMS fluctuations (RMSF) were calculated for the carbon alpha atoms of the V3 loop residues from the ensemble of structures after superimposition of residues 296 to 330 (complete V3 loop, plain lines) or superimposition of residues 308 to 315 (V3 tip, dotted lines). [file 12977_2015_177_MOESM4_ESM.pdf]

## Additional file 6

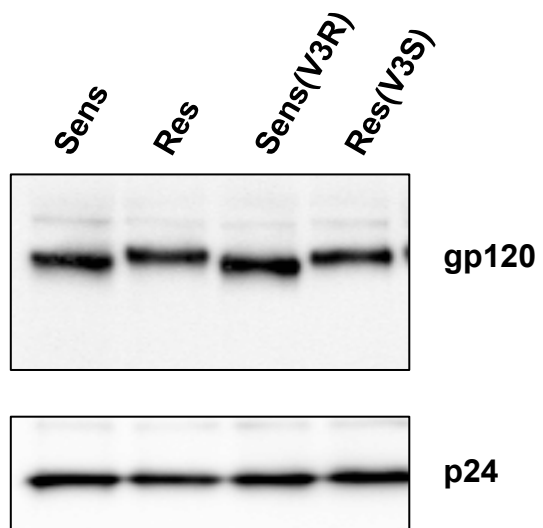

Supplement: Additional file 6: — Figure S4. Western blot analysis of gp120 and Gag p24 expression on MVC-Sens, MVC-Res, MVC-Sens(V3R) and MVC-Res(V3S) viral particles. Three hundred ng Gag p24 of the different viral isolates were solubilized in lysis buffer (100 mM (NH4)2SO4, 20 mM Tris-HCl (pH 7.5), 10 % glycerol, and 1% Triton X-100) and then loaded onto Biorad CriterionTM XT 4-12% Bis-Tris Gels under reducing conditions. Virus-associated gp120 and p24 were visualized using a sheep anti-HIV-1 gp120 polyclonal antibody (AALTO Bio Reagents LTD.) and a mouse anti-HIV-1 p24 monoclonal antibody (clone 183-H12-5C) (NIH AIDS Reagent Program), respectively, and then with the appropriate HRP-conjugated secondary antibodies (VECTOR). Bands were developed by enhanced chemiluminescence (Thermo Scientific) and were quantified using a LAS-1000 CCD camera (Image Gauge Software, Fuji Film Co., Tokyo, Japan). The figure shows that the viral isolates have gp120/p24 ratios R that are in the same range (R = 1, 1.15, 1.24 and 0.81 for MVC-Sens, MVC-Res, MVC-Sens(V3R) and MVC-Res(V3S), respectively). A representative experiment out of two is shown. [file 12977_2015_177_MOESM6_ESM.pdf]
