# Supplementary material for: A single-residue change in the HIV-1 V3 loop associated with maraviroc resistance impairs CCR5 binding affinity while increasing replicative capacity
Source: Retrovirology. 2015 Jun 18;12:50. doi: 10.1186/s12977-015-0177-1 (PMC4470041; doi:10.1186/s12977-015-0177-1)
Supplement: Additional file 3: — Supplemental text (related to Figure 7 and additional file 4). [file 12977_2015_177_MOESM3_ESM.pdf]

Additional file 3. Supplemental text (related to Figure 7 and additional file 4). The molecular dynamics simulations of the V3 loops from the MVC-Sens and MVC-Res isolates revealed significant conformational changes as a function of time. This was indicated by root mean square deviations relative to the mean structure approaching 4 Å and 5 Å for the V3 loops from MVC-Sens and MVC-Res, respectively (**Additional file 4a**). Furthermore, the distributions of the phi and psi torsion angles were broad and/or multimodal, revealing that the backbone of all residues including prolines experienced several conformational states. The atomic and angular fluctuations were calculated and averaged by residue for every 25 ps segment of the five molecular dynamics trajectories. The standard deviations of torsion angles averaged by residue were slightly but repeatedly smaller in the V3 tip of MVC-Res than in the V3 tip of MVC-Sens, suggesting that the Pro to Ser mutation and the Ala insertion have induced a slight decrease in the flexibility of the tip region (**Additional file 4b**). The average fluctuation of the C-alpha atoms in the V3 tip was around 3Å in the two variants (**Additional file 4c**), meaning that both the GPG and the GAPG motifs tend to adopt a preferential folded conformation. Previous structural studies showed that the tip of V3 consistently adopts a β-hairpin-like structure, with the GPG motif constituting the turn between the two anti-parallel strands [3,9,54]. Hydrogen bonds (H-bonds) established between backbone atoms of residues 308 to 315 were identified to characterize the structure of both V3 tips (**Figure 7**). Four H-bonds were present in 6 to 24% of the structures simulated for both MVC-Sens and MVC-Res V3 loops: one between residues 308 and 315, two between residues 310 and 313 and one in the turn between the backbone NH group of Arg-313 and the Pro-311 carbonyl group. Noteworthy, in 62% of the structures simulated for the MVC-Res V3 loop, an additional H-bond occurred between Gly-310 and Gly-312. In summary, models of both MVC-Res and MVC-Sens V3 loops revealed a tip organized from a turn centered on Pro-311. The insertion of Ala in MVC-Res induced the formation of a bulge in the turn, thereby altering the local conformation of the tip.
