# Supplementary material for: A single-residue change in the HIV-1 V3 loop associated with maraviroc resistance impairs CCR5 binding affinity while increasing replicative capacity
Source: Retrovirology. 2015 Jun 18;12:50. doi: 10.1186/s12977-015-0177-1 (PMC4470041; doi:10.1186/s12977-015-0177-1)
Supplement: Additional file 5: — Table S1. Intermolecular interactions between the V3 tips and CCR5. [file 12977_2015_177_MOESM5_ESM.pdf]

Additional file 5. Intermolecular interactions between the V3 tips and CCR5.

Intermolecular interaction: CCR5 atom(s) – V3 atom(s)	Occupancy rate of the interaction (%)	
	in CCR5 / MVC-Sens models	in CCR5 / MVC / MVC-Res models
**Ionic bond: TM7 E283 (COO^-) – R313 (guadinidium)**	63.3	0.0
**Ionic bond: TM7 D276 (COO^-) – R313 (guadinidium)**	22.5	30.0
**H-bond: ECL2 C178 (CO) – G312 (NH)**	65.0	0.0
**H-bond: ECL2 S179 (OH) – G310 (CO)**	75.8	0.0
**H-bond: ECL2 S180 (NH) – G312 (CO)**	99.2	0.0
**H-bond: ECL2 S180 (CO) – A314 (NH)[a]**	100.0	100.0
**H-bond: ECL2 F182 (NH) – A314 (CO)[a]**	25.8	92.5
**H-bond: ECL2 F182 (CO) – Y316 (NH)[a]**	2.5	39.2
**H-bond: N-terminus K26 (NH) – InsA (CO)**	not applicable	54.2
**π stacking: ECL2 F182 (phenyl) – F315 (phenyl)**	78.3	27.5
**π stacking: TM5 Y187 (phenyl) – F315 (phenyl)**	0.0	0.8

The occupancy rates of non covalent bonds were calculated for every 500 ps segment of the last 60 ns of molecular dynamics trajectories. An ionic bond was considered as occupied if the distance between the centroids of the two charged groups was lower than 5.5 Å. An hydrogen bond was considered as occupied if the distance between the donor and acceptor atoms was lower than 3.5 Å and the angle formed by the donor, hydrogen and acceptor atoms was higher than 100°. A π stacking was considered as occupied if the distance between the centroids of the two aromatic groups was lower than 4.5 Å.

[a] These H-bonds were initially enforced during the simulation, but corresponding restraints were removed during the last 60 ns.
