# Supplementary material for: A single-residue change in the HIV-1 V3 loop associated with maraviroc resistance impairs CCR5 binding affinity while increasing replicative capacity
Source: Retrovirology. 2015 Jun 18;12:50. doi: 10.1186/s12977-015-0177-1 (PMC4470041; doi:10.1186/s12977-015-0177-1)
Supplement: Additional file 7: — Figure S5. Molecular modeling of the complex between CCR5 and MVC-Sens V3. The graphic shows time series of RMSD values of the complex using as reference the input coordinates. The table indicates which constraints or restraints were imposed on the system during the simulation. bb : backbone atoms, all : all atoms, sc : side chains. [file 12977_2015_177_MOESM7_ESM.pdf]

Additional file 7

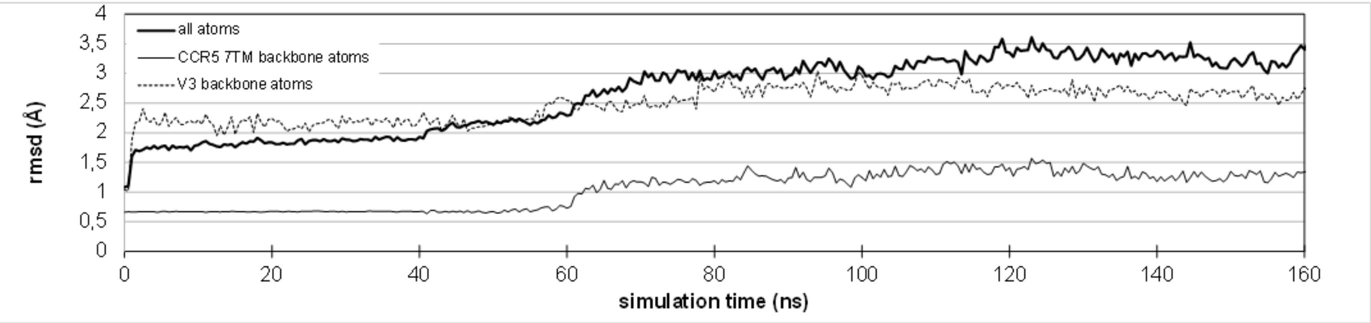

Time (ns)	1-20	20-40	40-60	60-80	80-100	100-160
**Contraints (10 kcal/mol.Å²)**						
**CCR5:** ECL2 & N-terminus						
**CCR5:** ICL1-3, ECL1, ECL3, C-terminus	all	bb				
**CCR5:** 7TM	all	all	bb			
**V3 tip:** residues P308-R313	all					
**V3 bridging sheet:** C296 & C330	bb	bb	bb	bb	bb	bb
**Restraints (distances and angles)**						
**CCR5 N-terminus – V3 base:**<br>Y10(OSO_3^-) – R326(guanidinium),<br>Y14(OSO_3^-) – R298(guanidinium), N302($CONH_2$), T303(OH)	sc	sc	sc	sc	sc	sc
**Intra V3 base:**<br>T297(NH) – H329(CO), T297(CO) – H329(NH)	bb	bb	bb	bb	bb	bb
**CCR5 TM7 – V3 tip:**<br>E283 (COO^-) – R313(guadinidium)	sc	sc	sc	sc	sc	
**CCR5 ECL2 – V3 tip:**<br>S180(CO) – A314(NH)<br>F182(CO) – Y316 (NH)<br>F182(NH) – A314(CO)	bb	bb	bb	bb	bb	
**Intra V3 tip:**<br>P308(CO) – F315(NH)	bb	bb	bb	bb		
