# Supplementary material for: A single-residue change in the HIV-1 V3 loop associated with maraviroc resistance impairs CCR5 binding affinity while increasing replicative capacity
Source: Retrovirology. 2015 Jun 18;12:50. doi: 10.1186/s12977-015-0177-1 (PMC4470041; doi:10.1186/s12977-015-0177-1)
Supplement: Additional file 8: — Figure S6. Molecular modeling of the complex between MVC-bound CCR5 and MVC-Res V3. The graphic shows time series of RMSD values of the complex using as reference the input coordinates. The table indicates which constraints or restraints were imposed on the system during the simulation. bb : backbone atoms, all : all atoms, sc : side chains. [file 12977_2015_177_MOESM8_ESM.pdf]

Additional file 8

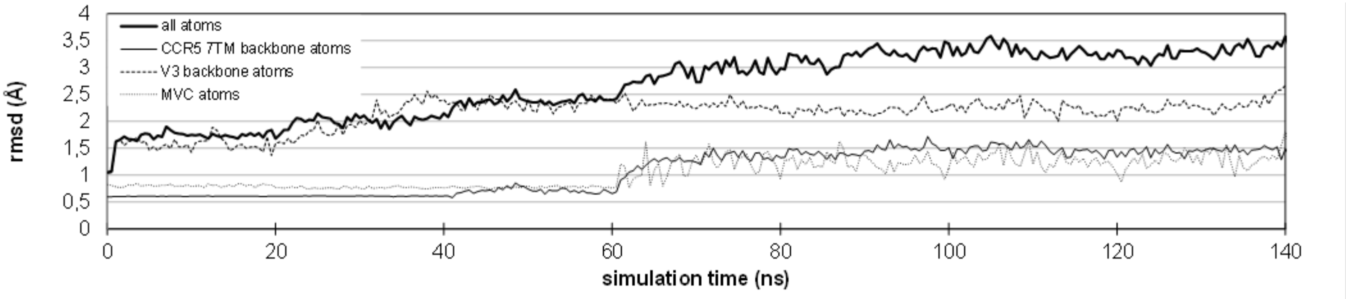

**Time (ns)**	**1-20**	**20-40**	**40-60**	**60-80**	**80-140**
**Contraints (10 kcal/mol.Å²)**					
**CCR5:** ICL1-3, ECL1, ECL3, C-terminus	all	bb			
**CCR5:** 7TM	all	all	bb		
**V3 tip:** residues S308-R313					
**V3 bridging sheet:** C296 & C330	bb	bb	bb	bb	bb
**MVC**	all	all	all		
**Restraints (distances and angles)**					
**CCR5 N-terminus – V3 base:**<br>Y10(OSO_3^-) – R326(guanidinium),<br>Y14(OSO_3^-) – R298(guanidinium), N302($CONH_2$), T303(OH)	sc	sc	sc	sc	sc
**Intra V3 base:**<br>T297(NH) – H329(CO), T297(CO) – H329(NH)	bb	bb	bb	bb	bb
**CCR5 TM7 – V3 tip:**<br>E283 (COO^-) – R313(guadinidium)					
**CCR5 ECL2 – V3 tip:**<br>S180(CO) – A314(NH)<br>F182(CO) – Y316 (NH)<br>F182(NH) – A314(CO)	bb	bb	bb	bb	
**Intra V3 tip:**<br>S308(NH) – F315(CO)<br>S308(CO) – F315(NH)	bb	bb	bb		
